# Therapeutic Effects of Intratracheally Nebulized Carnosine-Loaded Liposomes on Lipopolysaccharide-Induced Acute Lung Injury

**DOI:** 10.3390/pharmaceutics18091090

**Published:** 2026-08-29

**Authors:** Chao Fang, Lixin Xie, Daihan Xie, Jingting Yin, Shiji Zhang, Yiling Gan, Xiaodong Hou, Fanlei Kong, Yu Huo, Xiuli Liu

**Affiliations:** 1 School of Medicine, Tongji University, No. 500 Zhennan Road, Shanghai 200433, China; max999515@tongji.edu.cn (C.F.); xielixin415@163.com (L.X.); henritsie@163.com (D.X.); yinjingting2001@163.com (J.Y.); azh0410@126.com (S.Z.); ganyilng@email.sdfmu.edu.cn (Y.G.); hxd984@163.com (X.H.); 2 Department of Ultrasound in Medicine, Second Affiliated Hospital, School of Medicine, Zhejiang University, No. 88, Jiefang Road, Hangzhou 310009, China; kongfanlei@126.com; 3 Faculty of Chinese Medicine Science, Guangxi University of Chinese Medicine, No. 13 Wu-he Avenue, Nanning 530222, China; 4 Department of Medical Oncology, The First Affiliated Hospital of Guilin Medical University, No. 15 Le-qun Road, Guilin 541001, China

**Keywords:** carnosine, inhalation administration, acute lung injury, liposome, oxidative stress

## Abstract

**Introduction**: Acute lung injury (ALI) is characterized by severe inflammation and oxidative stress. However, effective pharmacological interventions for ALI remain limited. Carnosine, an endogenous dipeptide known for its redox-regulating and immunomodulatory activities, has demonstrated promising protective effects. In the present study, inhalable carnosine-loaded liposomes (Cn-Ls) were developed to enhance pulmonary delivery and achieve localized treatment in a lipopolysaccharide (LPS)-induced ALI model in mice. **Methods**: Cn-Ls were prepared and systematically evaluated for their morphology, stability, drug-loading capacity, and release kinetics. In vitro assays were performed to evaluate their cytocompatibility, antioxidant activity, and effects on LPS-induced reactive oxygen species (ROS) generation. In an ALI model, inhaled Cn-Ls were administered to assess pulmonary retention and therapeutic efficacy, including lung inflammation, oxidative stress, circulating levels of C-reactive protein (CRP), tumor necrosis factor (TNF)-α, interleukin (IL)-6, lung architecture, and respiratory function. **Results**: Encapsulation of carnosine within liposomes markedly prolonged its pulmonary retention (t_1_/_2_ = 1.7 h vs. 1.0 h for free carnosine), providing a more sustained lung-retentive delivery profile for up to 12 h. In vitro assays showed that Cn-Ls had excellent cytocompatibility, reduced cell death, exhibited potent antioxidant activity, and effectively suppressed LPS-induced ROS generation. In an ALI model, inhaled Cn-Ls markedly mitigated lung inflammation and oxidative stress, reduced circulating levels of CRP, TNF-α, and IL-6, preserved lung architecture, and improved respiratory function. Compared with free carnosine, Cn-Ls exhibited enhanced pulmonary retention and superior therapeutic efficacy. **Conclusions**: These results identified inhalable Cn-Ls as a potential nanotherapeutic approach for targeted ALI management and provided a foundation for further translational development. However, additional investigations are required to assess the long-term safety of Cn-Ls, optimize formulation stability and scalability, and further elucidate the underlying therapeutic mechanisms before clinical translation.

## 1. Introduction

Acute lung injury (ALI) is a severe respiratory disorder that can arise from diverse insults, such as infection, trauma, toxic exposure, and systemic inflammation [1,2,3,4]. ALI involves severe impairment of alveolar barrier integrity, accompanied by excessive inflammatory activation and oxidative stress. Clinically, ALI exhibits diverse manifestations and varying severity, with a substantial proportion of patients progressing to acute respiratory distress syndrome (ARDS), which is associated with high mortality. [5,6]. Although substantial progress has been made in elucidating the mechanisms underlying ALI, clinically effective treatments remain insufficient [7,8,9,10]. Thus, identifying novel intervention strategies for ALI is an important focus in both basic research and clinical practice.

The current management of ALI remains largely supportive, primarily relying on strategies such as ventilatory support and fluid therapy [11,12,13]. However, these strategies fail to directly target the mechanisms underlying oxidative stress and inflammation. Accordingly, the development of pharmacological approaches that can modulate these processes has emerged as a promising research direction. Carnosine is an endogenous bioactive dipeptide formed by β-alanine and L-histidine, exhibiting potent antioxidant and anti-inflammatory activities. It can scavenge ROS, inhibit lipid peroxidation, alleviate inflammation, and regulate apoptosis [14,15,16]. Accumulating evidence suggests that carnosine exerts protective activity in several models of oxidative stress-related disorders [17,18]. However, its therapeutic application in pulmonary diseases is hindered by its rapid metabolism, broad distribution, and low bioavailability [19,20]. To address these limitations, novel drug-delivery strategies such as liposome-based systems have attracted considerable interest.

**Figure 1:**
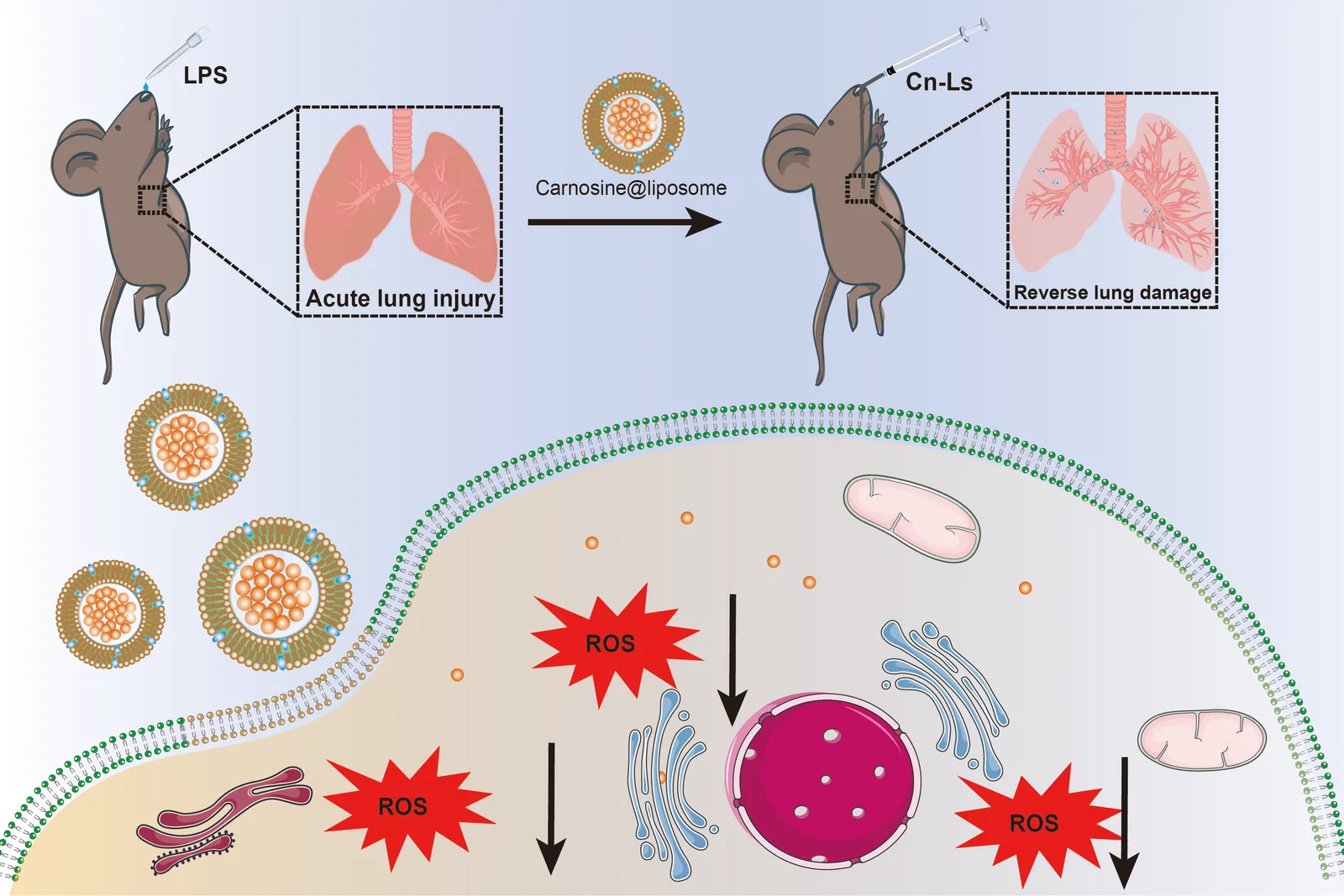
Cn-Ls mitigate LPS-induced ALI in mice. ALI was induced through intranasal infusion of LPS, which caused elevated ROS levels in lung tissue. Cn-Ls were delivered by aerosol inhalation, achieving even pulmonary distribution, reducing ROS, and mitigating LPS-induced injury.

Inhalation delivery offers unique advantages for the treatment of pulmonary diseases, including localized targeting, rapid onset of action, and fewer systemic side effects [21,22,23,24]. Nanotechnology can further improve the stability and targeted efficiency of inhaled formulations. Among various nanocarriers, liposomes are widely utilized due to their favorable biological compatibility, structural stability, and ability to provide sustained drug release [25,26,27]. On the basis of these advantages, we developed inhalable Cn-Ls to enhance the pulmonary delivery of carnosine and prolong its therapeutic effects. Using a mouse model of LPS-induced ALI that recapitulated the inflammatory and oxidative stress features of the disease, we investigated the therapeutic efficacy and underlying mechanisms of Cn-Ls (Figure 1) [28,29,30]. This study aimed to perform a preclinical evaluation of inhaled Cn-Ls for the treatment of LPS-induced ALI and investigate their therapeutic effects and underlying mechanisms.

## 2. Materials and Methods

### 2.1. Reagents and Cells

Dimethyl sulfoxide (DMSO), ethyl alcohol, phosphate-buffered saline (PBS), Cy5.5 (Cyanine 5.5 fluorescent dye), dipalmitoylphosphatidylcholine (DPPC), and DSPE-PEG2000-NHS (1,2-distearoyl-sn-glycero-3-phosphoethanolamine-N-[poly(ethylene glycol)2000]-N-hydroxysuccinimide) were purchased from Shanghai Aladdin Biochemical Technology Co., Ltd. (Shanghai, China). Carnosine and FITC-labeled anti-mouse Ly6G antibody were purchased from Sigma-Aldrich (St. Louis, MO, USA). Enzyme-linked immunosorbent assay (ELISA) kits for C-reactive protein (CRP), tumor necrosis factor (TNF)-α, interleukin (IL)-6, IL-10, monocyte chemoattractant protein (MCP)-1, cyclooxygenase-2 (COX-2), as well as the calcein/propidium iodide (PI) detection kit, 4-amino-5-methylamino-2′,7′-difluorofluorescein diacetate (DAF-FM DA; a nitric oxide fluorescent probe), ROS assay kit, BSA, anti-fluorescence quenching mounting medium, alanine aminotransferase (ALT), aspartate aminotransferase (AST), alkaline phosphatase (ALP), total protein (TP) Activity Detection Kit, and cell counting kit-8 (CCK-8) were purchased from Beyotime Biotechnology Co., Ltd. (Shanghai, China). The tissue fixative, 0.2% Triton X-100, and normal goat serum were purchased from Beijing Solarbio (Beijing, China). Mouse Creatinine (CREA) ELISA Kit and Blood Urea Nitrogen (BUN) ELISA Kit, produced by Wuhan Xin Jie Qi Technology Co., Ltd (Wuhan, China). All biochemical reagents were used without further purification. The RAW264.7 and MLE-12 cell lines were purchased from ATCC and cultured in Dulbecco’s modified Eagle’s medium supplemented with 10% fetal bovine serum, 100 U/mL penicillin, and 100 µg/mL streptomycin. All other chemicals were purchased from Aladdin Biochemical Technology Co., Ltd. (Shanghai, China).

### 2.2. Animals

Four-week-old female BALB/c mice were supplied by Slack Laboratory Animals Co., Ltd. (Shanghai, China) and housed under specific pathogen-free (SPF) conditions for one week prior to the experiments, with unrestricted availability of food and water. All animal experiments were approved by the Committee on Animals of the Guangxi University of Chinese Medicine. At the experimental endpoints, mice were humanely euthanized via CO_2_ inhalation followed by cervical dislocation.

### 2.3. Synthesis of Cn-Ls

Precisely weigh 10 mg of carnosine and dissolve it in 5 mL of double-distilled water. Additionally, precisely weigh 15 mg of dipalmitoyl phosphatidylcholine and 30 mg of DSPE-PEG2000-NHS, and place them in a 100 mL conical round-bottom flask. Add 6 mL of a mixture of chromatographic-grade chloroform and 3 mL of methanol. Sonicate at room temperature (40 kHz, 100 W) to aid dissolution for 5 min until the lipids are completely dissolved. Then, add the above carnosine aqueous solution drop by drop to the lipid organic solution, with the dripping rate controlled at 1 mL/min. Stir vigorously with a magnetic stirrer (300 rpm) while dripping to form an O/W type emulsion. Transfer the round-bottom flask to a rotary evaporator (Büchi R-300) and set the water bath temperature to 37 °C, vacuum degree to 0.1 MPa, rotation speed to 150 rpm. Continue rotary evaporation for 30 min until complete removal of the organic solvents, resulting in the formation of a uniform transparent lipid layer along the flask surface. After film formation, increase the vacuum degree to 0.1 MPa and continue to vacuum for 15 min to completely remove the residual organic solvents. Then, add 10 mL of phosphate buffer solution preheated to 45 °C in a 45 °C water bath and magnetically stir with a 200 rpm magnetic stirrer for 60 min to hydrate the lipid film and form a white lipidic suspension. Pass the hydrated lipidic suspension through polycarbonate filters with defined pore sizes of 200 nm and 100 nm each, 10 times using a lipidic extruder (Avanti Mini-Extruder, Avanti Polar Lipids, Alabaster, AL, USA) under room temperature conditions and a nitrogen pressure of 1.0 MPa. To remove the free freeform carnosine, take 2 mL of the extruded lipidic suspension and place it in an ultrafiltration centrifuge tube (retention molecular weight 100 kDa, Millipore Amicon Ultra, Millipore Corporation, Billerica, MA, USA) and centrifuge at 4000× *g* for 20 min. Discard the filtrate containing the free drug and resuspend the retained lipids with 2 mL of PBS. Repeat the above ultrafiltration washing operation 3 times. Finally, the collected Cn-Ls were dispersed in 2 mL PBS and maintained at 4 °C until further experiments. The encapsulation rate is calculated by measuring the carnosine content in the solution before and after ultrafiltration (HPLC), and the drug loading (%) = (encapsulated carnosine mass/total lipid mass) × 100%.

### 2.4. Characterization

The physicochemical characteristics of Cn-Ls, covering structural features, size distribution, surface charge, encapsulation capacity, and release profile, were evaluated using the homogeneous liposomal suspension before nebulization. These measurements represent the properties of the liquid-phase formulation and do not directly reflect potential changes that may occur after nebulization.

#### 2.4.1. Transmission Electron Microscopy

Transmission electron microscopy (TEM) imaging was performed to characterize the morphological features of Cn-Ls. The liposomal suspension was diluted with ultrapure water to achieve a final concentration of approximately 0.5 mg/mL and briefly centrifuged to remove visible large aggregates during TEM sample preparation. A drop (5 μL) of the diluted sample was placed onto a plasma-treated carbon-coated copper grid (300 mesh) and incubated for 60 s to allow sample adsorption. Excess liquid was removed, and the samples were subsequently subjected to negative staining using 2% (*w*/*v*) phosphotungstic acid for 60 s. Following air drying at room temperature, TEM images were collected using a field-emission TEM (Thermofisher, Waltham, MA, USA) operated at 120 kV under low-dose conditions.

#### 2.4.2. Particle Size and Zeta Potential of Cn-Ls

The hydrodynamic diameters and zeta potentials of the Cn-Ls were measured using a Malvern Zetasizer (ZEN3600, Malvern Instruments Ltd., Malvern, Worcestershire, UK). The samples were diluted with ion-free ultrapure water to approximately 0.5 mg/mL and gently sonicated before measurement to ensure uniform dispersion of the liposomal suspension. Particle size measurements were performed at 25 °C, with 150 scans per run and three independent measurements. Zeta potential was determined using a folded capillary cell at 25 °C, and three independent measurements were obtained for each sample. Data are presented as mean ± standard deviation.

#### 2.4.3. Parameters of the Aerosol Aerodynamic Particle Size Distribution Measured by APS

The diluted liquid samples are placed in the atomizer, which atomizes them into stable aerosols through compressed air. After removing excess moisture through the drying tube, the aerosols are introduced into the sample inlet of the APS through the gas conduit to ensure stable airflow and matching flow rate with the instrument’s set sampling flow rate. Each sample is typically collected for 3 min until sufficient particle counts are obtained to ensure statistical representativeness. After each measurement, the particle size distribution histogram, mass median aerodynamic diameter (MMAD), and geometric standard deviation (GSD) parameters of the sample are recorded. After completing each sample, the atomizer and the sampling pipeline are cleaned with blank solvent or clean air to eliminate cross-contamination. During the entire experiment, the environmental temperature and humidity need to be recorded regularly and background subtraction should be performed promptly.

#### 2.4.4. Ultraviolet Detection of Carnosine

The carnosine concentration was determined using a UV-Vis spectrophotometer (Agilent Cary 3500, Agilent Technologies, Inc., Santa Clara, CA, USA). Standard carnosine solutions were generated through stepwise dilution in deionized water to achieve final concentrations of 0.3, 0.1, 0.03, 0.01, and 0.003 mg/mL. Absorbance was measured using a 1 cm quartz cuvette at 25 °C, with deionized water as the blank. Full-spectrum scans were recorded over the range of 200–600 nm to identify the characteristic absorption peaks. Each concentration was measured in triplicate. A standard calibration curve was constructed using the linear regression of absorbance versus concentration.

#### 2.4.5. HPLC Detection of Carnosine

Precisely weigh an appropriate amount of the muscle peptide standard substance, dissolve it in pure water and prepare an appropriate concentration standard stock solution. Then, perform stepwise dilution to generate five standard working solutions with different concentrations. Take each concentration standard solution and react it with the derivatizing reagent 2,4-dinitrofluorobenzene (DNFB) under alkaline conditions for a column-preceding derivatization reaction. After the reaction is completed, filter it through a 0.22 μm microporous membrane and measure it using a high-performance liquid chromatograph equipped with an ultraviolet detector. The chromatographic conditions are as follows: the chromatographic column is selected as Kromasil NH2 column (4.6 mm × 200 mm, 5 μm, Kromasil, Eka Chemicals AB, Bohus, Sweden), the mobile phase is a mixture of acetonitrile and 0.15 mol/L sodium acetate buffer solution (volume ratio 1:5), the flow rate is 1.0 mL/min, the column temperature is 35 °C, the detection wavelength is 360 nm, and the injection volume is 10–25 μL. Record the chromatographic peak area of each concentration standard solution, plot the standard curve with peak area as the ordinate and the corresponding standard solution concentration as the abscissa, and perform linear regression fitting to obtain the regression equation. For the detection of muscle peptide in liposomes, take an appropriate amount of liposome samples, add an appropriate amount of methanol to disrupt the liposome structure to release the encapsulated muscle peptide, then add pure water, extract by ultrasonic for 30 min, centrifuge at 3000 r/min for 10 min, and take the supernatant for filtration through a 0.22 μm microporous membrane. Treat the filtrate with the same derivatization method as the standard solution, analyze it under the same chromatographic conditions, and record the sample chromatographic peak area. Substitute the sample peak area into the above standard curve regression equation to calculate the content of muscle peptide in liposomes.

#### 2.4.6. In Vitro Release of Carnosine from Cn-Ls

The in vitro release behavior of carnosine in Cn-Ls was evaluated by dialysis method. A total of 2 mL of the Cn-L suspension containing a known amount of carnosine was precisely measured and placed in a dialysis bag (with a molecular weight cut-off of 100 kDa, Spectra/Por, Repligen Corporation, Rancho Dominguez, CA, USA) that had been soaked in deionized water for 12 h. The bag was sealed and immersed in a release medium containing 20 mL of phosphate buffer solution. The samples were maintained in the dark at 37 °C under continuous shaking at 100 rpm using a temperature-controlled incubator. At predetermined intervals (1, 2, 4, 8, and 12 h), 1.0 mL aliquots of the release medium were collected and placed into 1.5 mL centrifuge tubes. It was centrifuged at 10,000× *g* for 5 min at 4 °C, and the supernatant was filtered through a 0.22 μm microporous membrane. The carnosine concentration was determined by high-performance liquid chromatography (HPLC). Meanwhile, an equal volume (1.0 mL) of freshly heated fresh PBS was added to the release system to maintain a constant volume. The HPLC system was equipped with a C18 reversed-phase column (ZORBAX Eclipse Plus C18, 4.6 × 250 mm, 5 μm, Agilent Technologies, Inc., Santa Clara, CA, USA). The mobile phase was methanol: phosphate buffer (20 mmol/L potassium dihydrogen phosphate, pH 3.0) = 5:95 (*v*/*v*). It was eluted isothermally, with a flow rate of 1.0 mL/min. The detection wavelength was 230 nm (determined by scanning the pre-experiment to identify the characteristic absorption peak of carnosine), the column temperature was 30 °C, the injection volume was 20 μL, and the run time was 15 min. A series of concentration standard solutions were prepared using carnosine standard substances (Sigma-Aldrich) under the above chromatographic conditions. A standard curve was plotted under these conditions, and the carnosine in the samples was quantified using the external standard method. All samples were measured in triplicate and a blank PBS was set as a negative control.

### 2.5. Cell Experiments

#### 2.5.1. Cell Viability Assay

MLE-12 cells at the exponential growth stage were plated into 96-well plates at 1 × 10^3^ cells/well and cultured for 24 h to facilitate cell adhesion. Subsequently, the original medium was removed and replaced with fresh medium containing different treatments (Control, LPS, Carnosine, and Cn-Ls). After 24 h of incubation, 10 μL of Cell Counting Kit-8 (CCK-8) reagent was introduced into each well, and the plates were further incubated for 2 h. The absorbance values were quantified at 450 nm with a microplate spectrophotometer. In addition, 50 μM hydrogen peroxide was selected as the positive control group. Logarithmic growth phase MLE-12 cells were taken and inoculated at a density of 1 × 10^3^ cells per well in a 96-well plate. Each well was supplemented with 100 µL of complete medium, while PBS was added to the peripheral wells to minimize edge effects. The plate was placed in the incubator for pre-culture for 24 h. The next day, after observing that the cells adhered well, the original medium was discarded, and the experiment was divided into four groups: control group, hydrogen peroxide group, Cn-L group, and hydrogen peroxide + Cn-L group. After adding the drugs, the cells were placed in the incubator to continue culturing for 24 h. Following the incubation period, 10 µL of CCK-8 solution was introduced into each well, and the plate was gently agitated to ensure uniform mixing. Then, it was incubated in a 37 °C, 5% CO_2_ incubator in the dark for 2 h. The absorbance at 450 nm of each well was measured using a multi-functional microplate reader. For each condition, five technical replicates were analyzed, and the experiment was independently repeated three times. Data were presented as mean ± standard deviation. Based on the cell viability results, the optimal concentration for subsequent in vitro experiments was determined to be 100 μg/mL. The cell viability remained well above 90% at 10 and 50 μg/mL. As the concentration increased to 100 μg/mL, the cell viability was still approximately 80%, indicating excellent biocompatibility within this range. Although the survival rate decreased slightly at 500 and 1000 μg/mL (dropping to approximately 75–78%), the 100 μg/mL concentration was selected as it provides a desirable balance between maintaining high cell viability (>80%) and ensuring sufficient pharmacological dosage for subsequent efficacy assays (such as anti-inflammatory or cellular uptake studies). Additionally, considering the high local concentration characteristics of inhalation administration, 100 μg/mL serves as an ideal non-toxic dose capable of exerting therapeutic potential without significant cytotoxicity.

#### 2.5.2. Live/Dead Staining Assay

Cell viability and cytotoxicity were evaluated by dual staining with calcein-AM and propidium iodide (PI). MLE-12 cells were plated into 24-well plates and allowed to attach for 24 h before undergoing Control, LPS, Carnosine, or Cn-L treatments. After treatment, the cells were washed with PBS and incubated with calcein-AM/PI staining solution at 37 °C in the dark for 30 min. Following incubation, the cells were washed with PBS and immediately observed under a laser confocal microscope. Green (calcein-AM; excitation [Ex]/emission [Em] = 494/517 nm) and red fluorescence (PI; Ex/Em = 535/617 nm) were recorded. The experiments were independently repeated three times.

#### 2.5.3. Intracellular ROS Detection

Intracellular ROS levels were measured using the 2′,7′-dichlorofluorescin diacetate (DCFH-DA) fluorescent probe. RAW264.7 cells in the logarithmic growth phase were seeded in confocal dishes and underwent Control, LPS, Carnosine, or Cn-L treatment. After treatment, cells were washed twice with prewarmed PBS and incubated with DCFH-DA (10 μM, diluted in serum-free medium) at 37 °C for 30 min in the dark. The cells were then washed three times with PBS to remove excess probe and replenished with fresh culture medium. Fluorescence images were acquired using a Zeiss LSM 880 confocal laser scanning microscope (Carl Zeiss AG, Oberkochen, Germany) equipped with ZEN software (ZEN 3.0). Images were captured at 40× objective lens (Ex/Em = 488/525 nm). To ensure unbiased comparison, identical imaging acquisition parameters (including laser power, pinhole size, gain, and scan speed) were strictly maintained across all experimental groups.

#### 2.5.4. Intracellular NO Detection

Intracellular nitric oxide (NO) levels were quantified using the fluorescent indicator DAF-FM DA. RAW264.7 cells were seeded in confocal dishes and underwent Control, LPS, Carnosine, or Cn-L treatment. After treatment, the cells were washed with PBS and incubated with DAF-FM DA (5 μM, serum-free medium) at 37 °C for 30 min in the dark. The cells were subsequently washed and incubated with fresh complete medium for 30 min to allow probe de-esterification. Fluorescence images were acquired using a Zeiss LSM 880 confocal laser scanning microscope equipped with ZEN software. Images were captured at 40× objective lens (Ex/Em = 495/515 nm). To ensure unbiased comparison, identical imaging acquisition parameters (including laser power, pinhole size, gain, and scan speed) were strictly maintained across all experimental groups. All experiments were independently repeated three times.

### 2.6. Animal Experiments

For biosafety evaluation, healthy mice were randomly assigned to receive Cn-Ls (100 μg per mouse) via tail vein injection. Major organs, including the heart, liver, spleen, lungs, and kidneys, were harvested for histological analysis by hematoxylin and eosin (H&E) staining.

To establish the LPS-induced ALI model, mice were anesthetized by an intraperitoneal injection of pentobarbital sodium (50 mg/kg). After achieving adequate anesthesia, ALI was induced by intranasal instillation of LPS (10 μg per mouse) dissolved in sterile saline. Mice were maintained in a supine position during the procedure and placed on a heating pad during recovery to prevent hypothermia. For therapeutic intervention, free carnosine or Cn-Ls were administered to the lungs via intratracheal nebulization following endotracheal intubation using a micro-spray aerosolizer (Bojian Biotechnology Co., Ltd., Shanghai, China). This device utilizes a compressed-air-driven atomization mechanism to convert the liquid formulation into a fine mist through a specialized micro-nozzle. According to the manufacturer’s specifications, the generated aerosol droplets have a mass median aerodynamic diameter (MMAD) of approximately 15 μm, which facilitates efficient deposition in the bronchoalveolar region while minimizing upper airway impaction. The nebulizer maintains a stable output rate of 50 μL per actuation, with an extremely low residual volume to guarantee precise and reproducible dosing. Each mouse received 50 μL of free carnosine solution or Cn-L suspension (100 μg/mL), corresponding to a dose of 5 μg per mouse. To evaluate the efficacy of different delivery routes, a separate cohort of mice received Cn-Ls via tail vein injection for kinetic ROS analysis at 3, 6, 9, and 12 h. The body temperature and respiratory rate were continuously monitored throughout the experimental period to assess physiological status and treatment response.

#### 2.6.1. H&E Staining

Histological alterations in major organs, including the heart, liver, spleen, lung, and kidney tissues, were examined using H&E staining. At the completion of the experiment, mice were sacrificed, and major organs were collected immediately and immersed in 4% paraformaldehyde for 24 h. The fixed tissues were processed by sequential dehydration in graded ethanol solutions, xylene clearing, paraffin embedding, and sectioning at 5 μm thickness. Following paraffin removal and rehydration, tissue sections underwent H&E staining, followed by dehydration, clearing, and mounting with neutral resin.

#### 2.6.2. Dihydroethidium Staining

Superoxide production in the lung tissues was assessed by dihydroethidium (DHE) fluorescence staining. Fresh lung specimens were collected, washed with ice-cold PBS, embedded in optimal cutting temperature (OCT) compound, and immediately cryopreserved in liquid nitrogen. Frozen sections (10 μm) were prepared and incubated with DHE working solution (10 μM in PBS) at 37 °C for 30 min in the dark. After washing with PBS, the sections were mounted with an antifade mounting medium containing 4′,6-diamidino-2-phenylindole (DAPI) and immediately imaged using a laser confocal microscope. DHE fluorescence was detected at Ex/Em wavelengths of 518/605 nm, and DAPI fluorescence was detected at Ex/Em wavelengths of 358/461 nm.

#### 2.6.3. Ly6G Staining

Immediately immerse freshly extracted mouse lung tissue in 4% paraformaldehyde fixative solution and fix it at 4 °C overnight. Then, embed it in paraffin and make paraffin sections. Place the sections on glass slides, wash them with PBS 3 times, each time for 5 min, and then treat them with 0.2% Triton X-100 at room temperature for 15 min for permeabilization. Wash them again with PBS. Next, add the blocking solution containing 5% BSA and 5% normal goat serum at room temperature for 1 h. After blocking, directly add the fluorescently labeled anti-mouse Ly6G antibody (1:200) and incubate it at 4 °C in a humid box under light overnight. After incubation, wash 3 times with PBS, each time for 5 min, then add the DAPI staining solution, re-stain at room temperature under light for 10 min, and finally gently wash away the excess DAPI with PBS and add the anti-fluorescence quenching mounting agent and cover with a cover glass for mounting. Observe under a confocal microscope. The green fluorescence signal represents Ly6G-positive neutrophils, and the blue fluorescence signal represents the cell nucleus.

#### 2.6.4. Cell Count in BALF

To detect the percentage and count of macrophages, neutrophils, lymphocytes and eosinophils in the bronchoalveolar lavage fluid of mice, the following experimental procedure is adopted: First, perform bronchoalveolar lavage on the experimental animals to collect the lavage fluid. The specific operation is to euthanize the mice, expose their tracheas and insert a lavage needle or catheter, and rinse them with pre-cooled sterile PBS. For bilateral lungs, use 1 mL of PBS for repeated rinsing 3 times. The recovered liquid is the bronchoalveolar lavage fluid. The collected bronchoalveolar lavage fluid is centrifuged at 1500 rpm for 10 min at 4 °C, resuspended in 1 mL of PBS containing 1% BSA, and 10 μL of the resuspended solution is taken for total cell counting using a hemocytometer under a microscope. After completing the total cell count, take an appropriate amount of the remaining cell suspension, centrifuge again, and retain an appropriate amount of the supernatant to resuspend the cells. The resuspended cells are spread onto a glass slide using a cell centrifuge slide apparatus and dried naturally on the slide. Then, the slide is placed in 10% neutral formaldehyde solution for fixation for more than 10 min. After staining, the cells can be classified and counted under an optical microscope based on their morphological characteristics, classified as macrophages, neutrophils, lymphocytes and eosinophils, and the number of each type of cell is recorded and its percentage of the total count of cells is calculated. Then, combined with the previously measured total cell count, the absolute count of each type of cell in each milliliter of lavage fluid can be calculated.

#### 2.6.5. The Content of Carnosine in the Lungs of Mice Was Detected Using HPLC Method

A measured quantity of mouse lung tissue was weighed and homogenized in pre-cooled phosphate buffer solution at a tissue-to-buffer ratio of 0.1 g/mL. The sample was thoroughly homogenized at low temperature using a tissue homogenizer. Then, protein precipitation was performed to remove large molecular interfering substances from the sample. A total of 9 times the volume of acetonitrile was introduced into the sample, and it was left to act at 4 °C for 10 min. The precipitated samples were centrifuged at 14,000 rpm for 10 min at 4 °C. The resulting supernatants were filtered using a 0.22 μm membrane filter and transferred into sample vials for injection. Chromatographic separation was achieved on an HILIC column under optimized conditions. The column was operated at 40 °C with gradient elution using 0.1% formic acid in water as mobile phase A and acetonitrile as mobile phase B at a flow rate of 250 μL/min. Detection was performed using a mass spectrometer equipped with an electrospray ionization source operated in multiple reaction monitoring mode. The carnosine concentration in mouse lung tissue was determined by substituting the peak area ratio of carnosine to the internal standard into the calibration curve equation.

#### 2.6.6. Organ Fluorescence Imaging

Cy5.5 fluorescent dye-labeled Cn-Ls and the same molar concentration of free Cy5.5 were respectively administered to the lungs of mice through an atomization device. At 3, 6, and 12 h following nebulization, mice from each group were sacrificed by cervical dislocation, and the heart, liver, spleen, lung, and kidney tissues were promptly collected for further analysis. The surface residual blood was washed off with pre-cooled physiological saline, and then the samples were dried with filter paper and placed on the sample stage of the in vivo fluorescence imaging system. Using a Cy5.5-specific excitation filter (excitation wavelength 640–680 nm) and emission filter (emission wavelength 690–750 nm), fluorescence imaging of each isolated organ was performed under the same parameters. After image collection, the average fluorescence intensity was analyzed using the software provided by the imaging system to evaluate the biological distribution and metabolism of the Cy5.5 marker in different organs.

#### 2.6.7. Measurement of Lung Wet Weight Ratio of Mice

The mice were euthanized by intraperitoneal injection of pentobarbital sodium or cervical dislocation. The thoracic cavity was quickly opened, and the bilateral lung tissues were completely removed along with the trachea and surrounding connective tissues. The surface blood residues were washed off with pre-cooled phosphate-buffered saline, and the surface moisture was gently absorbed using filter paper. The wet weight of the lung tissues was immediately measured using a precision electronic balance. Following determination of the wet weight, lung tissues were wrapped in pre-weighed aluminum foil and dried in an oven at 80 °C for 48 h until a stable dry weight was obtained. The dry weight was then measured again, and the lung wet-to-dry weight ratio (W/D ratio) = wet weight/dry weight was calculated.

#### 2.6.8. Hematological and Biochemical Analysis

At the designated time points, whole blood was obtained from the orbital venous plexus. For systemic safety evaluation, collected blood samples were immediately placed into EDTA-K2 anticoagulation tubes for hematological assessment. Parameters including hemoglobin (HGB), red blood cell (RBC) count, platelet (PLT) count, and white blood cell (WBC) count were quantified using an automated hematology analyzer.

Serum levels of CRP, TNF-α, IL-6, and IL-10 were quantified using ELISA kits (Abcam plc, Cambridge, UK) according to the manufacturers’ instructions. For biochemical analysis, collected blood samples were incubated at room temperature to allow coagulation, followed by centrifugation at 3000× *g* for 15 min at 4 °C to separate serum. Serum absorbance was determined at 450 nm using an ELISA plate reader, and cytokine levels were quantified according to the corresponding standard curves. Each sample was measured in three technical replicates.

#### 2.6.9. Measurement of MCP-1 Levels in Bronchoalveolar Lavage Fluid

The concentration of MCP-1 in bronchoalveolar lavage fluid (BALF) was determined using an ELISA kit. Following euthanasia, the trachea was carefully isolated and intubated, and bronchoalveolar lavage was performed by flushing the lungs three times with ice-cold sterile PBS. The recovered BALF samples were centrifuged at 500× *g* for 10 min at 4 °C, and the resulting supernatants were collected for MCP-1 quantification according to the manufacturer’s protocol. Absorbance values were determined at 450 nm using a microplate reader, and MCP-1 concentrations were calculated based on the corresponding calibration curve. Each sample was analyzed in duplicate.

#### 2.6.10. Liver and Kidney Biochemical Tests

The tests are conducted using ELISA kits. First, the mouse serum samples and a series of standard samples are added to the enzyme-labeled plate wells that have been coated with corresponding antibodies. The samples are incubated to allow the antigens to bind with the antibodies. The plates are washed, followed by the addition of HRP-labeled detection antibodies for another incubation. After the washing steps, TMB substrate was introduced for color development under light-protected conditions, followed by the addition of stop solution to terminate the reaction. The absorbance of each well is measured at 450 nm using an enzyme analyzer. The content of the corresponding indicators in the samples is calculated based on the standard curve. The specific operation is as follows: 100 μL of substrate buffer and 20 μL of the sample are added to a 96-well microplate, mixed and incubated at 37 °C for 15 min. Then, 80 μL of reaction termination solution is added, mixed and measured at 405 nm using an enzyme analyzer. Simultaneously, blank and standard samples are tested. The activity of each indicator is calculated based on the standard curve.

## 3. Results

### 3.1. Synthesis and Characterization of Cn-Ls

**Figure 2:**
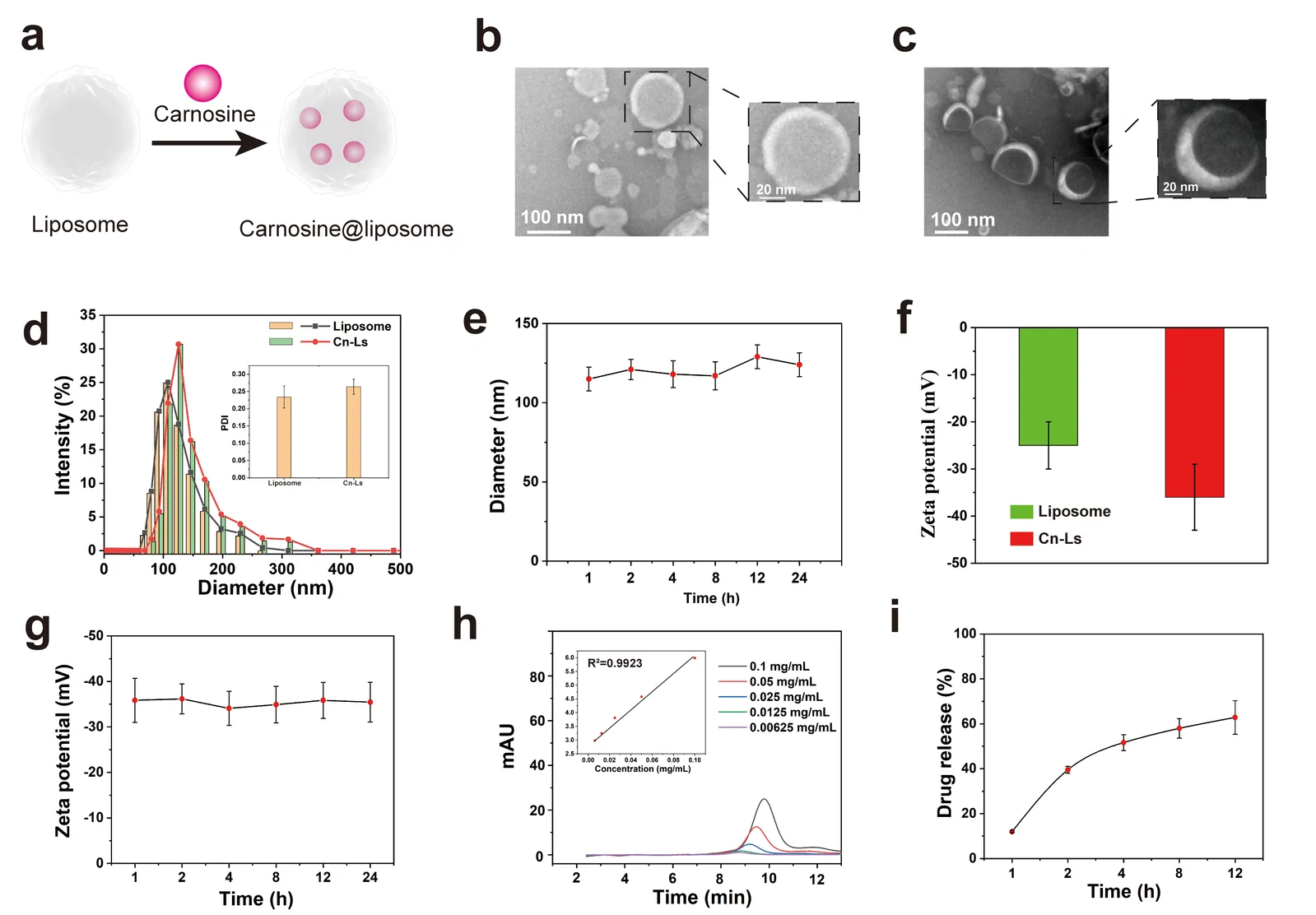
(**a**) Schematic diagram of Cn-L synthesis. (**b**) TEM image of liposome (scale bar: 100 nm). (**c**) TEM image of Cn-Ls (scale bar: 100 nm). (**d**) Particle size and PDI index of liposomes and Cn-Ls measured by a nanoparticle size analyzer. Data are expressed as mean ± SD (*n* = 3). (**e**) Particle size changes in Cn-Ls within 1 to 24 h. Data are expressed as mean ± SD (*n* = 3). (**f**) Zeta potential of liposomes and Cn-Ls. Data are expressed as mean ± SD (*n* = 3). (**g**) Time-dependent changes in the zeta potential of Cn-Ls during storage. Data are expressed as mean ± SD (*n* = 3). (**h**) HPLC chromatograms of carnosine. (**i**) Release profile of carnosine in Cn-Ls at different time points. Data are expressed as mean ± SD (*n* = 3).

Figure 2a shows a schematic flow diagram of carnosine encapsulation within liposomes. TEM images were acquired to evaluate the morphology and size of the liposomes, which revealed a mean diameter of approximately 120 nm (Figure 2b). The incorporation of carnosine did not markedly alter the particle size, since the Cn-Ls maintained a similar diameter (~120 nm, Figure 2c). Dynamic light scattering (DLS) analysis further confirmed that both blank liposomes and Cn-Ls exhibited particle sizes within the range of 100–150 nm, in agreement with TEM observations, and polydispersity indices (PDIs) between 0.2 and 0.3, indicative of a uniform size distribution (Figure 2d). A stability assessment demonstrated that the particle size of the Cn-Ls remained unchanged over 24 h, indicating good structural stability (Figure 2e). To further evaluate the aerosolization performance of the formulations for pulmonary delivery, the aerodynamic particle size distribution was measured using an Aerodynamic Particle Sizer (APS). As presented in Table 1, both blank liposomes and Cn-Ls exhibited mass median aerodynamic diameters (MMADs) of 14.47 ± 2.43 µm and 14.86 ± 3.74 µm, respectively, with geometric standard deviations (GSDs) of 1.25 ± 0.21 and 1.32 ± 0.18. These results suggest that the encapsulation of carnosine did not significantly affect the aerosolization properties, and the MMAD of approximately 15 µm is highly suitable for efficient lung deposition via intratracheal nebulization. Zeta potential measurements showed a lower surface potential of Cn-Ls than blank liposomes (Figure 2f), which may be associated with carnosine encapsulation. To facilitate calculation of carnosine loading in the liposomes, we determined the ultraviolet absorption spectra of carnosine at different concentrations (Supplementary Figure S1). The stability of the surface charge of Cn-Ls over time was further evaluated by monitoring changes in zeta potential (Figure 2g). To quantify carnosine and validate the analytical method, HPLC analysis was performed using a series of standard concentrations. As displayed in Figure 2h, the representative chromatograms showed that carnosine eluted with a retention time of approximately 9.5–10.0 min without any interfering peaks. The inset of Figure 2h displays the calibration curve established from the relationship between carnosine concentration and peak area. A strong linear relationship was observed across the concentration range of 0.00625 to 0.1 mg/mL. Using this method, Cn-Ls at a concentration of 20 mg/mL achieved a drug-loading efficiency of 20.2% (Figure 2h). This established a reliable and sensitive quantitative method for determining carnosine content in subsequent formulations. Moreover, in vitro release studies demonstrated sustained release of carnosine from Cn-Ls extending up to 12 h (Figure 2i), providing the foundation for their potential application in the treatment of lung injury.

### 3.2. Cell Experiments

**Figure 3:**
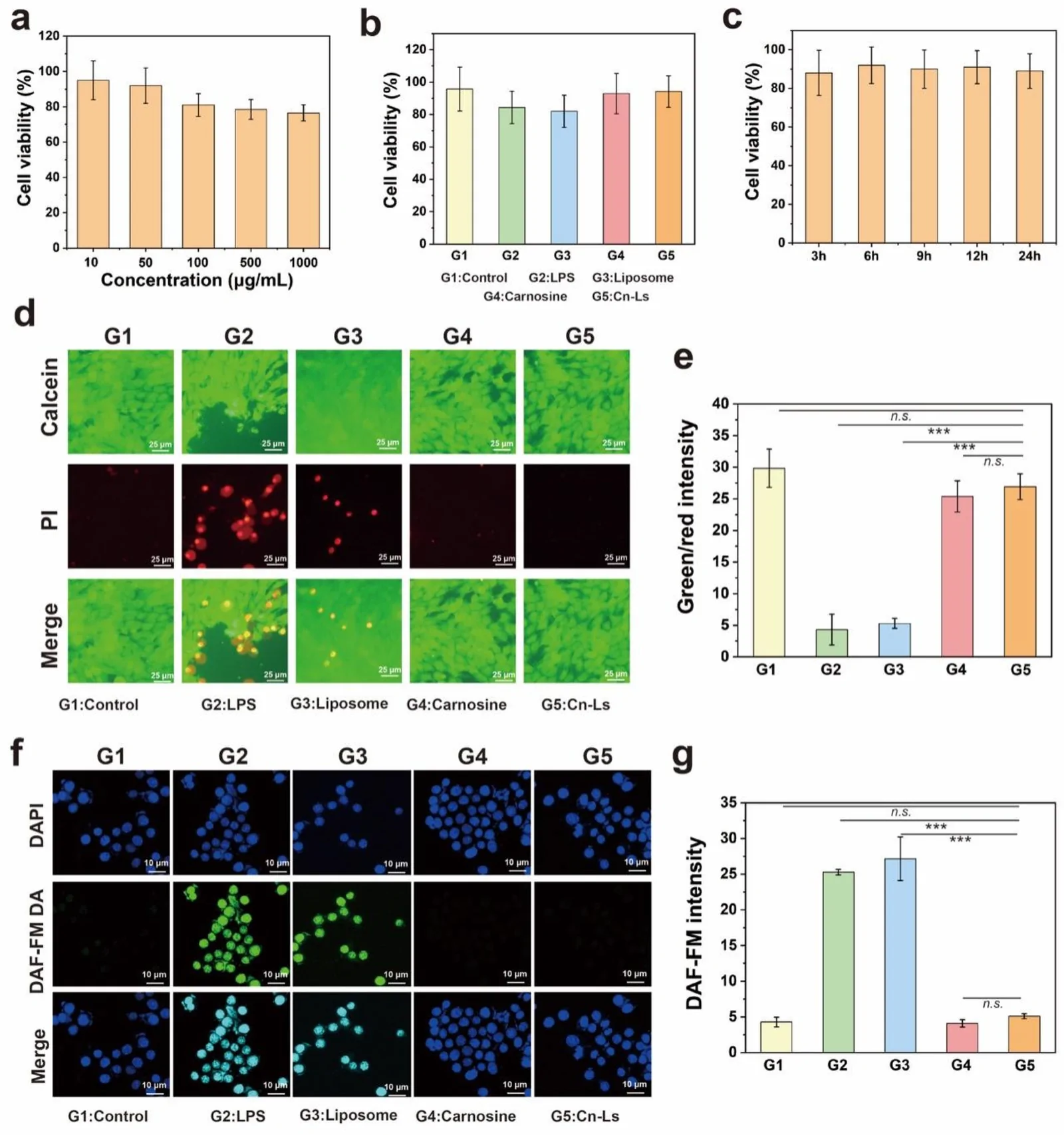
(**a**) Viability of MLE-12 cells treated with different concentrations of Cn-Ls. Data are expressed as mean ± SD (n = 3). (**b**) Viability of MLE-12 cells treated with different materials. Data are expressed as mean ± SD (n = 3). (**c**) Viability of MLE-12 cells treated with Cn-Ls for different durations. Data are expressed as mean ± SD (n = 3). (**d**) Live (green)/dead (red) staining of MLE-12 cells treated with different materials (scale bar: 25 μm). The maximum excitation wavelength of calcein is 494 nm, and the maximum emission wavelength is 517 nm. The maximum excitation wavelength of the PI complex is 535 nm, and the maximum emission wavelength is 617 nm. (**e**) Quantification of green/red fluorescence intensity in different groups. Data are expressed as mean ± SD (n = 3). Significance was analyzed using one-way ANOVA with Tukey’s post hoc test for multiple comparisons; *** *p* < 0.001, n.s. indicates not significant. (**f**) CLSM images of NO after different experimental treatments in RAW264.7, scale bar: 10 μm. Note: G1–G4 represent Control, LPS, LPS + Carnosine, LPS + Cn-Ls. (**g**) Quantification of DAF-FM intensity in different groups. Data are expressed as mean ± SD (n = 3). Significance was analyzed using one-way ANOVA with Tukey’s post hoc test for multiple comparisons; *** *p* < 0.001, n.s. indicates not significant.

The biosafety of the nanocarriers was first assessed to ensure their suitability for subsequent experiments. Cell viability was determined using the CCK-8 assay (Figure 3a–c and Figure S2). To further verify cytocompatibility, calcein/PI staining was performed after LPS stimulation. Only the LPS-treated group showed noticeable cell death, whereas minimal cytotoxicity was detected in the other groups (Figure 3d,e) [31]. Furthermore, after RAW264.7 cells underwent different treatments, notable NO production was observed in the cells treated with LPS, while the Cn-L-treated group exhibited significantly reduced NO levels compared with the LPS-treated group (Figure 3f,g).

**Figure 4:**
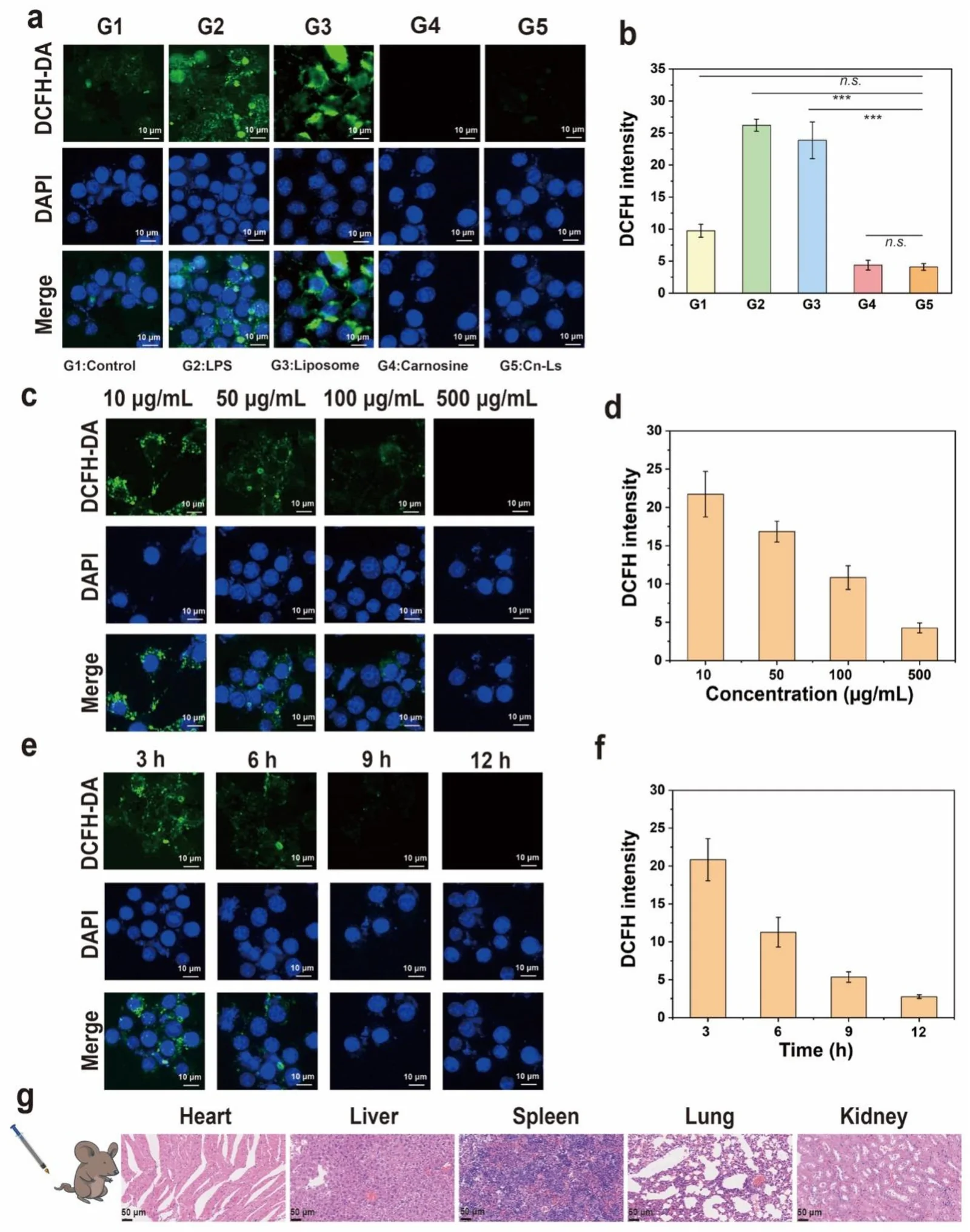
(**a**) CLSM images of ROS levels in RAW264.7 cells treated with different materials (scale bar: 10 μm). (**b**) Quantification of DCFH intensity in different groups. Data are expressed as mean ± SD (*n* = 3). Significance was analyzed using one-way ANOVA with Tukey’s post hoc test for multiple comparisons; *** *p* < 0.001, n.s. indicates not significant. (**c**) CLSM images of ROS levels in RAW264.7 cells treated with different concentrations of Cn-Ls (scale bar: 10 μm). (**d**) Quantification of DCFH intensity at different concentrations. Data are expressed as mean ± SD (*n* = 3). (**e**) CLSM images of ROS levels in RAW264.7 cells treated with Cn-Ls for different durations (scale bar: 10 μm). DCFH-DA: excitation wavelength, 488 nm; emission wavelength, 525 nm. DAPI: maximum excitation wavelength, 358 nm; maximum emission wavelength, 461 nm. (**f**) Quantification of DCFH fluorescence intensity at different time points. Data are expressed as mean ± SD (*n* = 3). (**g**) H&E staining of major organs from mice after intravenous administration of Cn-Ls (scale bar: 50 μm). Note: G1–G4 represent Control, LPS, LPS + Carnosine, LPS + Cn-Ls.

To evaluate the protective effect of Cn-Ls on RAW264.7 cells, LPS was applied to induce oxidative stress. Confocal laser scanning microscopy (CLSM) revealed markedly increased intracellular ROS levels in the LPS group, as indicated by enhanced green fluorescence, whereas no obvious ROS signal was observed in the carnosine or Cn-L groups (Figure 4a,b). Analysis across varying Cn-L concentrations and exposure durations demonstrated a negative correlation between intracellular ROS levels and both parameters, further supporting the protective capacity of Cn-Ls against LPS-induced oxidative stress (Figure 4c–f). In addition, to evaluate the biosafety of Cn-Ls in mice, we injected 100 μg of Cn-Ls into the tail vein of the mice. H&E staining of the major organs revealed no histopathological abnormalities, confirming the systemic safety of Cn-Ls and supporting their suitability for subsequent pulmonary inhalation therapy (Figure 4g).

### 3.3. Animal Experiments

**Figure 5:**
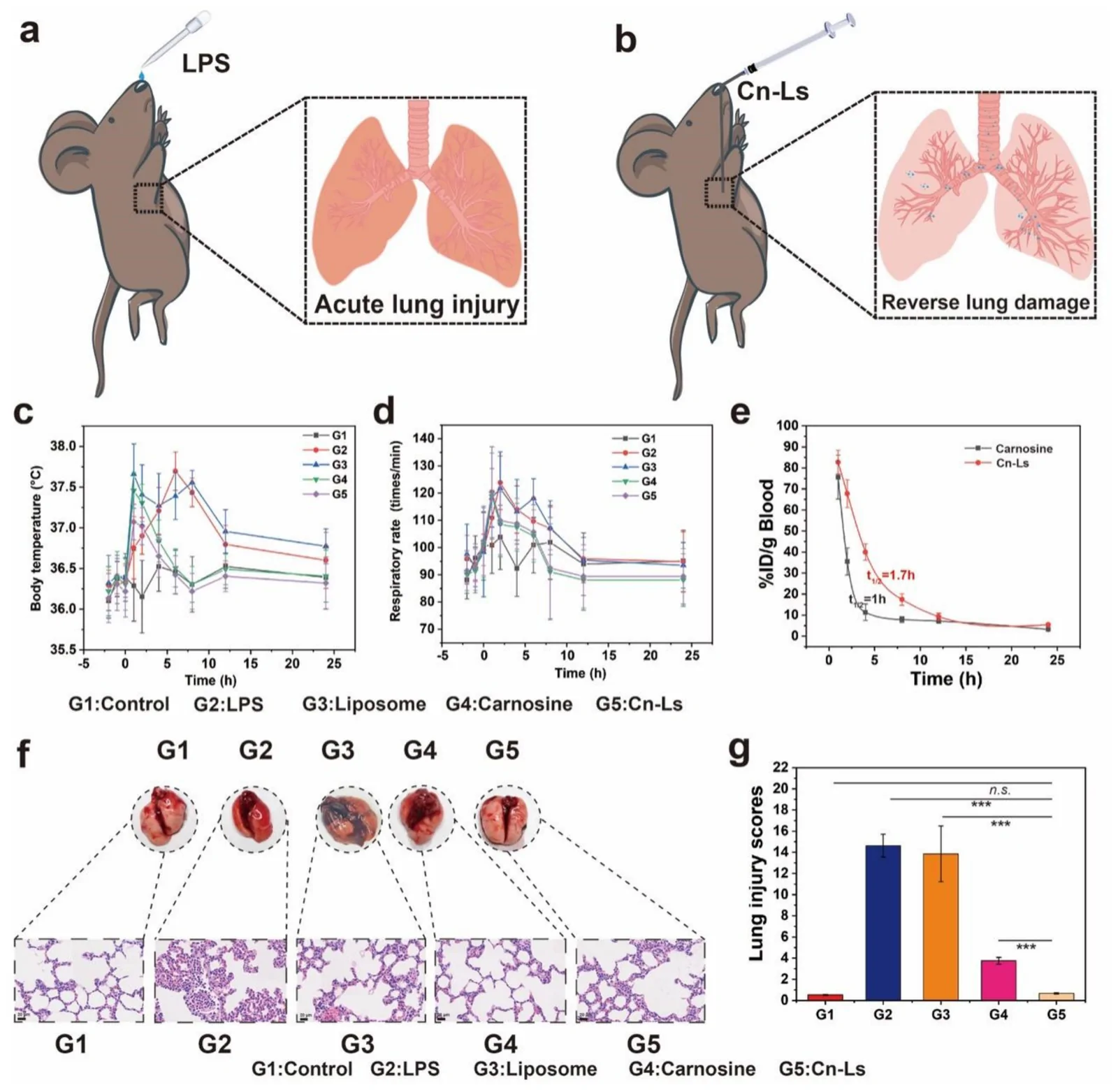
(**a**) Schematic representation of ALI induction by intranasal LPS administration in mice. (**b**) Schematic showing that inhaled Cn-Ls mitigated LPS-induced ALI in mice. (**c**) Body temperature of mice treated with different materials. Data are expressed as mean ± SD (*n* = 3). (**d**) Respiratory rate of mice treated with different materials. Data are expressed as mean ± SD (*n* = 3). (**e**) Pharmacokinetic detection of Cn-Ls and carnosine in mouse blood. Data are expressed as mean ± SD (*n* = 3). (**f**) Representative H&E-stained lung sections from different treatment groups (scale bar: 20 μm). (**g**) Blinded histopathological lung injury scoring. Note: G1–G4 represent Control, LPS, LPS + Carnosine, LPS + Cn-Ls. Data are expressed as mean ± SD (*n* = 5). Significance was analyzed using one-way ANOVA with Tukey’s post hoc test for multiple comparisons; *** *p* < 0.001, n.s. indicates not significant.

In vivo experiments were conducted to assess the therapeutic potential of Cn-Ls in a murine model of ALI. After anesthesia, mice were intranasally administered LPS to induce ALI (Figure 5a). To ensure a uniform distribution, the animals were maintained in an upright position with gentle vertical rotation. For treatment, carnosine or Cn-Ls were delivered to the lungs via intratracheal nebulization following endotracheal intubation (Figure 5b). Physiological monitoring revealed an obvious increase in body temperature following LPS administration, indicating a systemic inflammatory response consistent with the induction of ALI. However, mice treated with carnosine or Cn-Ls exhibited a more rapid reduction in body temperature than the mice in the LPS group (Figure 5c). Respiratory rate analysis revealed that Cn-L treatment partially restored the LPS-induced alterations in respiratory patterns, suggesting an improvement in respiratory status and further supporting the protective effects of Cn-Ls (Figure 5d). Pharmacokinetic evaluation demonstrated that liposomal encapsulation prolonged the systemic circulation time, thereby enhancing the therapeutic availability of carnosine (Figure 5e). To further evaluate lung injury at the structural level, representative H&E-stained lung sections were analyzed (Figure 5f). Severe alveolar destruction and inflammatory cell infiltration were observed in the LPS group, whereas both carnosine- and Cn-L-treated groups showed markedly preserved lung architecture. Consistently, blinded histopathological scoring further confirmed significantly higher lung injury scores in the LPS group and markedly reduced scores in all treatment groups (Figure 5g). In addition, time-course histological analysis in the Cn-L-treated group demonstrated progressive attenuation of inflammatory injury over time (Supplementary Figure S3). Lung edema was further assessed by the wet/dry (W/D) weight ratio, which was significantly increased in the LPS group but markedly reduced in all treatment groups (Supplementary Figure S5). To evaluate systemic inflammatory responses and safety, serum cytokine levels including CRP, TNF-α, IL-6, and IL-10 were measured at multiple time points (12, 24, 48, and 72 h), showing no significant changes following Cn-L treatment (Supplementary Figure S7). In addition, hematological parameters including HGB, RBC, PLT, and WBC remained stable across all groups and time points (Supplementary Figure S8).

**Figure 6:**
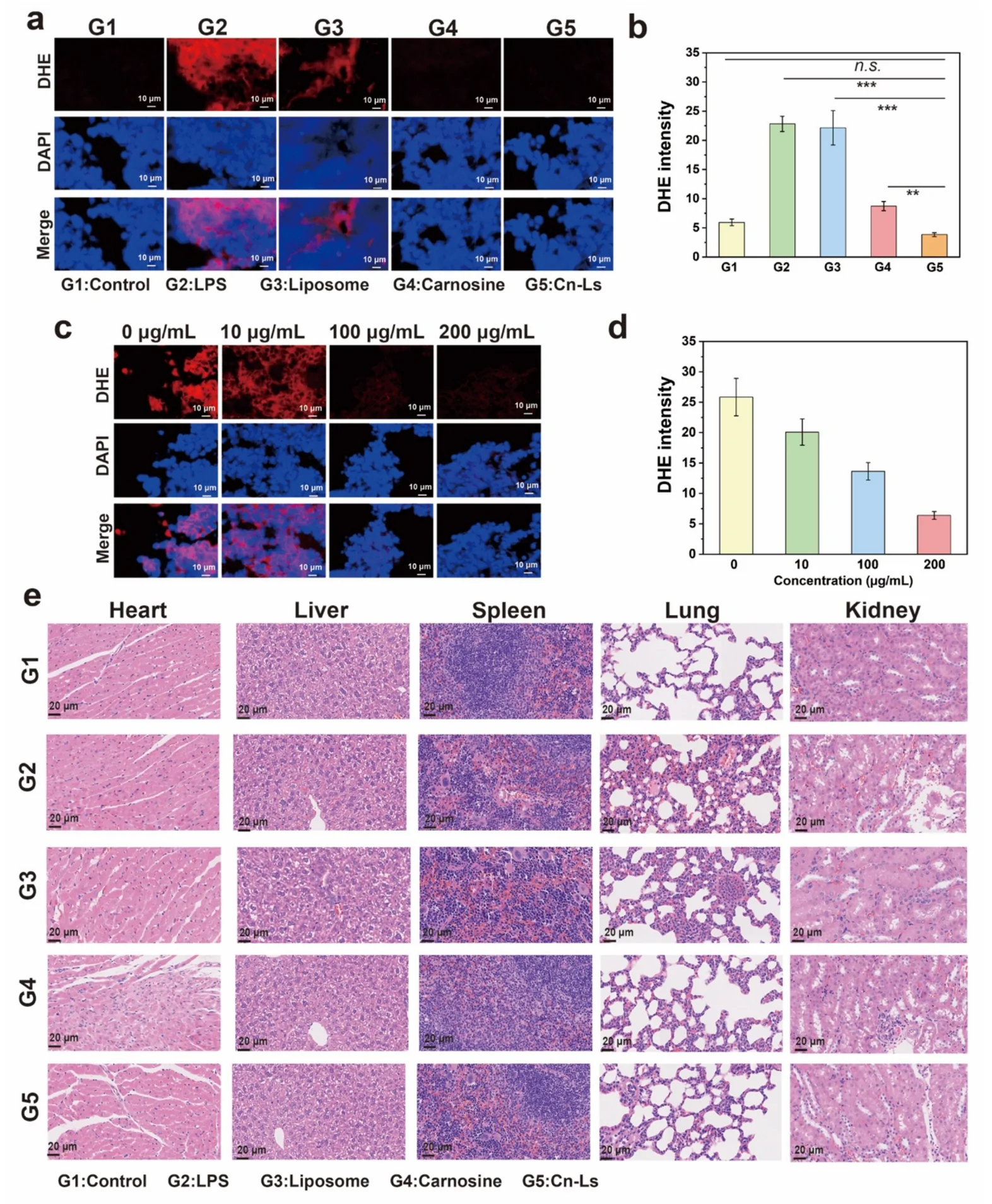
(**a**) CLSM images of ROS levels in lung tissues of ALI mice treated with different materials (scale bar: 10 μm). (**b**) Quantification of DHE intensity in different groups. Data are expressed as mean ± SD (*n* = 3), ** *p* < 0.01 and *** *p* < 0.001, n.s. indicates not significant. (**c**) CLSM images of ROS levels in lung tissues of ALI mice treated with Cn-Ls for different durations (scale bar: 10 μm). DHE: maximum excitation wavelength, 535 nm; maximum emission wavelength, 610 nm. DAPI: maximum excitation wavelength, 358 nm; maximum emission wavelength, 461 nm. (**d**) Quantification of DHE intensity at different concentrations. Data are expressed as mean ± SD (*n* = 3). (**e**) H&E staining of major organs from ALI mice after inhalation of different materials (scale bar: 20 μm). Note: G1–G4 represent Control, LPS, LPS + Carnosine, LPS + Cn-Ls.

DHE staining of the lung tissues revealed marked ROS accumulation in the LPS group, whereas the carnosine and Cn-L treatment groups exhibited markedly reduced ROS signals, demonstrating effective ROS scavenging (Figure 6a,b). Treatment with different concentrations of Cn-Ls further attenuated the progression of ALI in a dose-dependent manner (Figure 6c,d). H&E staining revealed pronounced pulmonary edema in the LPS group, whereas the lung architecture in the Cn-L group remained largely intact, confirming the protective role of Cn-Ls against tissue damage (Figure 6e).

**Figure 7:**
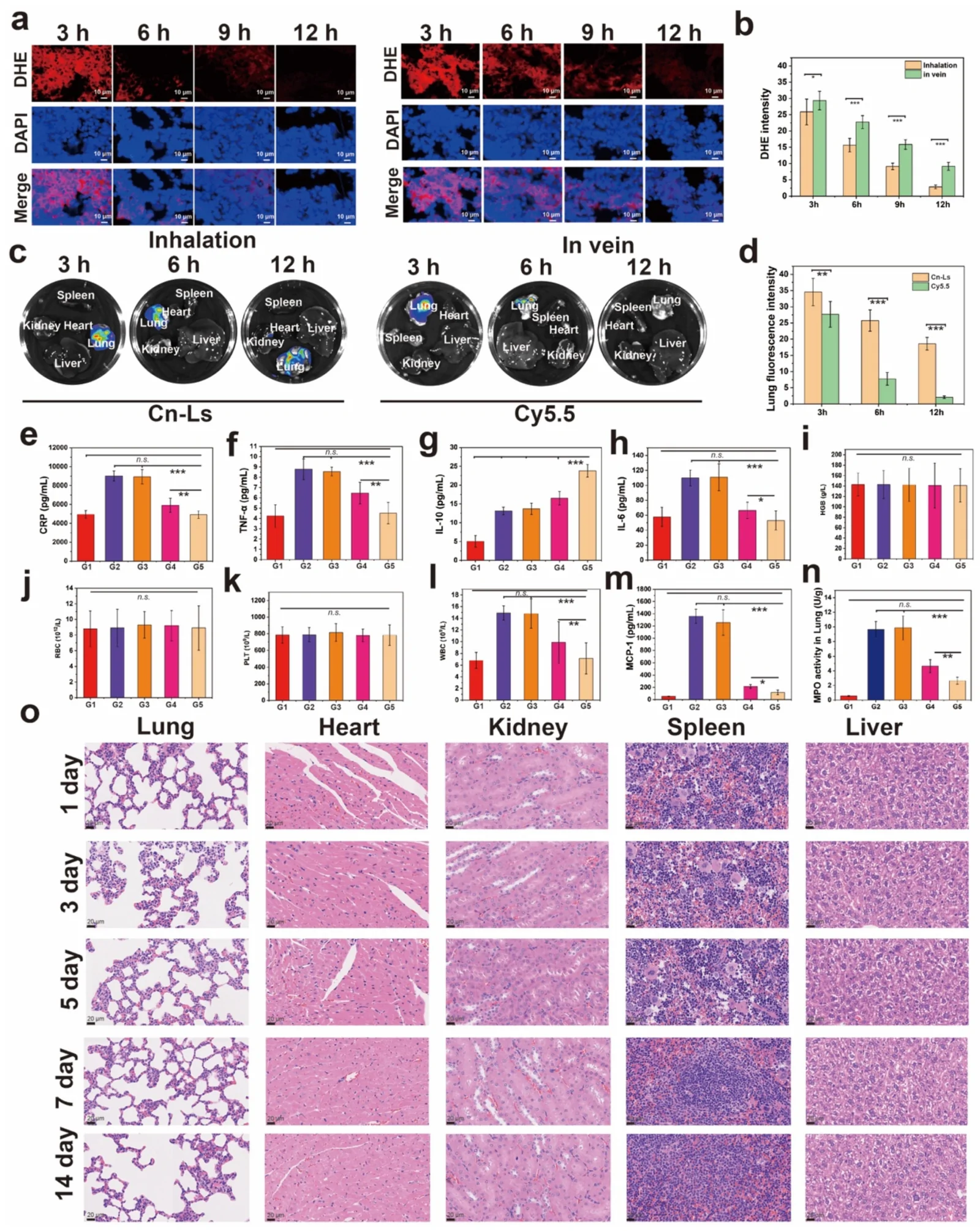
(**a**) CLSM images of ROS levels in lung tissues following Cn-Ls administration by inhalation or tail vein injection at different time points (scale bar: 10 μm). DHE: maximum excitation wavelength, 535 nm; maximum emission wavelength, 610 nm. DAPI: maximum excitation wavelength, 358 nm; maximum emission wavelength, 461 nm. (**b**) Quantification of DHE fluorescence intensity at different time points. Data are expressed as mean ± SD (*n* = 3). Significance was analyzed using one-way ANOVA with Tukey’s post hoc test for multiple comparisons; * *p* < 0.05, ** *p* < 0.01 and *** *p* < 0.001. (**c**) In vivo fluorescence imaging showing the biodistribution of Cy5.5-labeled Cn-Ls or free Cy5.5 in major organs (including heart, liver, spleen, lung, and kidney) at different time points (3, 6, and 12 h). Cy5.5: maximum excitation wavelength, 675 nm; maximum emission wavelength, 690 nm. (**d**) Quantitative analysis of fluorescence intensity in lung tissues over time. Data are expressed as mean ± SD (n = 3). Significance was analyzed using one-way ANOVA with Tukey’s post hoc test for multiple comparisons. (**e**) CRP, (**f**) TNF-α, (**g**) IL-10, (**h**) IL-6 levels in ALI mice treated with different materials. (**i**) Blood HGB levels in ALI mice treated with different materials. Blood (**j**) RBC, (**k**) PLT, and (**l**) WBC counts in ALI mice treated with different materials. (**m**) Expression of MCP-1 in the lung tissues of mice after different experimental treatments. (**n**) MPO activity in the lung tissues of mice after different experimental treatments. Data are expressed as mean ± SD (*n* = 3). Note: G1–G5 represent Control, LPS, Liposome, LPS + Carnosine, LPS + Cn-Ls. (**o**) H&E images of various organs at 1 day, 3 days, and 5 days post-inhalation of Cn-Ls into mouse lungs (scale bar: 20 μm). Significance was analyzed using one-way ANOVA with Tukey’s post hoc test for multiple comparisons; * *p* < 0.05, ** *p* < 0.01 and *** *p* < 0.001, n.s. indicates not significant.

To compare the therapeutic effects of intrapulmonary and caudal vein administration, both methods were applied to mouse models. Intrapulmonary delivery of Cn-Ls showed superior ROS-scavenging efficacy than intravenous administration of free carnosine, likely due to direct lung targeting, prolonged drug retention, and reduced systemic clearance (Figure 7a,b) [32,33]. To further verify lung distribution and retention, in vivo fluorescence imaging was performed using Cy5.5-labeled Cn-Ls and free Cy5.5. As shown, Cn-Ls exhibited sustained fluorescence signals in the lung up to 12 h, whereas free Cy5.5 showed rapid clearance with detectable signal only at 3 h (Figure 7c). Quantitative analysis of lung fluorescence intensity further confirmed significantly enhanced pulmonary retention of Cn-Ls compared with free dye (Figure 7d). Changes in the blood levels of inflammatory factors are significant indicators of therapeutic efficacy. ELISA was employed to measure the levels of pro-inflammatory factors, including CRP (Figure 7e), TNF-α (Figure 7f), and IL-6 (Figure 7h), as well as the anti-inflammatory cytokine IL-10 (Figure 7g). The levels of pro-inflammatory markers were significantly elevated in the LPS group but were markedly reduced following treatment with carnosine or Cn-Ls. Conversely, the level of IL-10 was further upregulated in the Cn-L-treated group (Figure 7g), suggesting an enhanced anti-inflammatory response. Given that IL-10 is a key anti-inflammatory cytokine involved in maintaining immune homeostasis and suppressing excessive inflammatory responses, the increased IL-10 production may contribute to the protective effects of Cn-Ls by promoting the resolution of inflammation. To further evaluate pulmonary inflammatory responses, MCP-1 levels in the bronchoalveolar lavage fluid were measured (Figure 7m). MCP-1 secretion increased markedly in the LPS group, whereas both carnosine and Cn-L treatments significantly suppressed its production. To further characterize inflammatory cell infiltration in the lung, differential immune cell populations in bronchoalveolar lavage fluid were analyzed. Compared with the LPS group, Cn-L treatment significantly reduced the proportions of neutrophils and eosinophils, while alterations in macrophage and lymphocyte populations were also observed (Supplementary Figure S6). These findings further support the immunomodulatory effects of Cn-Ls by reducing inflammatory cell recruitment in the pulmonary microenvironment. In addition, MPO activity in lung tissues, which was assessed by ELISA (Figure 7m), was significantly downregulated in the Cn-L-treated group in comparison with the LPS group, further confirming the anti-inflammatory effects of Cn-Ls at the tissue level. To further assess the biosafety of the Cn-Ls, blood tests were conducted for hemoglobin (HGB) (Figure 7i), red blood cells (RBCs) (Figure 7j), platelets (PLT) (Figure 7k), and white blood cells (WBCs) (Figure 7l). Among the hematological parameters examined, the WBC count was markedly elevated in the LPS group. In contrast, no significant changes were observed in the other components. Furthermore, serum biochemical analyses were performed to evaluate potential hepatic and renal toxicity following Cn-L administration. Liver function-related parameters, including alanine aminotransferase (ALT), aspartate aminotransferase (AST), alkaline phosphatase (ALP), and total protein (TP), as well as renal function-related parameters, including blood urea nitrogen (BUN) and creatinine (CREA), showed no significant abnormalities after Cn-L treatment (Supplementary Figures S9 and S10). Additionally, histological examination of organs from ALI mice administered Cn-Ls by pulmonary delivery on days 1, 3, and 5 revealed no evidence of Cn-L-induced damage, indicating the biological safety of Cn-Ls (Figure 7o). These findings underscore the safety of Cn-Ls and suggest their potential application in additional animal models for further validation.

## 4. Discussion

Inhalable nanocarrier platforms have emerged as an attractive approach for ALI therapy by enabling localized pulmonary delivery and reducing unwanted systemic distribution. In this study, we developed inhalable Cn-Ls and demonstrated their therapeutic potential in an LPS-induced ALI mouse model. Our results demonstrate that Cn-Ls mitigated lung inflammatory responses, attenuated oxidative damage, and enhanced pulmonary outcomes, highlighting their therapeutic potential for inflammatory lung disorders.

Compared with free carnosine, inhaled Cn-Ls exhibited improved therapeutic efficacy, which may be attributed to enhanced formulation stability, prolonged retention, and improved delivery efficiency provided by the liposomal carrier. Although free carnosine is effective in the treatment of ALI, its rapid metabolism and poor lung accumulation limit its clinical application [34]. After nano-modification, the liposome-encapsulation strategy enhances the pulmonary distribution of carnosine and enables its sustained release. Despite a drug-loading efficiency of 20.2%, Cn-Ls exhibited ROS-scavenging and anti-inflammatory effects comparable to those of free carnosine. Furthermore, the efficacy of these liposomes was superior to that of free carnosine administered intravenously, primarily because of their high targeting efficiency and favorable pulmonary retention. In comparison with traditional systemic administration, inhalation drug delivery offers distinct advantages in treating pulmonary diseases by directly targeting the affected site, reducing systemic side effects, and improving drug utilization [35,36]. The liposomes developed in this study exhibited a small particle size of approximately 120 nm, facilitating their penetration through the alveolar barrier and enhancing their local therapeutic effects [35]. In addition, liposome encapsulation enhances the stability and the local bioavailability and bio-retention of carnosine, thereby prolonging its residence time in lung tissue [37]. The lung mucus layer is mainly composed of negatively charged mucin, and positively charged nanoparticles tend to undergo electrostatic interactions with it, leading to entrapment in the mucus and rapid clearance by ciliary action. In contrast, negatively or neutrally charged nanoparticles more readily penetrate the mucus layer and reach the lower respiratory tract [38]. A previous study compared liposomes with different surface charge properties and demonstrated that anionic liposomes showed enhanced mucus penetration in an in vitro assay. In addition, negatively charged liposomes exhibited improved formulation stability and reduced cytotoxicity [39].

This study also systematically investigated the inflammation-regulating and antioxidative activities of carnosine in ALI. First, Cn-Ls reduced ROS levels in the lung tissue, indicating a strong inhibitory effect on oxidative stress. Second, Cn-Ls exerted anti-inflammatory effects by inhibiting the levels of pro-inflammatory factors. The levels of CRP, TNF-α, and IL-6 were lower in the Cn-L treatment group, highlighting the multi-targeted role of carnosine in modulating inflammation. In addition, carnosine may contribute to pulmonary recovery by maintaining alveolar structural integrity and supporting tissue barrier homeostasis [40,41]. The properties of carnosine observed in this study are consistent with those reported previously [40,41,42]. Notably, our study had two highlights: (1) Cn-L treatment was administered after LPS exposure, in accordance with clinical practice [41]. (2) To our knowledge, this study represents the first investigation of inhaled carnosine for the treatment of LPS-induced ALI accompanied by extensive safety evaluations.

However, this study had some limitations. First, although the efficacy of inhaled Cn-Ls was demonstrated in mouse models, further studies are required to evaluate their safety and effectiveness in larger animal models and clinical trials. Second, this study primarily focused on evaluating the therapeutic efficacy, pulmonary targeting capability, and biosafety of Cn-Ls. Although the antioxidant and anti-inflammatory effects were demonstrated through functional assessments, the specific molecular pathways underlying these effects remain to be elucidated. Future studies involving pathway-specific analyses, such as investigation of oxidative stress- and inflammation-related signaling pathways, will be necessary to further clarify the molecular mechanisms of Cn-L-mediated protection. Third, this study mainly focused on oxidative stress and inflammation, whereas other potential mechanisms, such as anti-apoptotic effects, were not thoroughly explored. Moreover, the long-term stability and scalability of Cn-Ls for industrial production require further optimization. In addition, although Cy5.5 fluorescence imaging provided valuable information regarding the biodistribution and pulmonary retention of Cn-Ls, this approach may not fully represent the in vivo pharmacokinetic behavior of released carnosine. Finally, the co-administration of peptides with other small molecules by inhalation has not been sufficiently studied.

## 5. Conclusions

In this study, we successfully developed inhalable carnosine liposomes using liposomal encapsulation and demonstrated their significant therapeutic effects in an LPS-induced ALI model. Cn-Ls markedly mitigated oxidative stress and inflammatory changes in lung tissue by scavenging ROS and inhibiting inflammatory responses. The innovative aspect of this study lies in the combination of inhalation delivery with a liposome carrier, which maximized the therapeutic effects and enhanced the efficiency of pulmonary drug delivery. In conclusion, the inhaled Cn-Ls developed in this study demonstrate promising potential for further preclinical development, paving a new avenue for the precision treatment of ALI and other oxidative stress-related disorders.

## Figures and Tables

**Table 1:** Parameters of the aerosol aerodynamic particle size distribution measured by APS.

	**MMAD (µm)**	**GSD**
Liposome	14.47 ± 2.43	1.25 ± 0.21
CN-Ls	14.86 ± 3.74	1.32 ± 0.18

## Data Availability

Data are contained within the article.

## Supplementary Materials

The following supporting information can be downloaded at: https://www.mdpi.com/article/10.3390/pharmaceutics18091090/s1. Figure S1. UV-Vis absorption spectra of carnosine at various concentrations (0.003, 0.01, 0.03, 0.1, and 0.3 mg/mL) measured over a wavelength range of 200–600 nm. (a) The left panel and its inset show the absorption profiles and the corresponding calibration curve illustrating the linear relationship between the absorbance and carnosine concentration. (b) The right panel displays a representative UV-Vis spectrum of carnosine, highlighting the characteristic absorption peak at approximately 210 nm. Figure S2. Viability of MLE-12 cells in different groups. Data are expressed as mean ± SD (*n* = 3). Significance was analyzed using one-way ANOVA with Tukey’s post hoc-test for multiple comparisons; and *** *p* < 0.001. Figure S3. Time-course H&E staining of lung tissues in the Cn-Ls-treated group at 3, 6, 9, and 12 h post-administration (scale bar: 20 μm). Figure S4. Carnosine levels in lung tissues of different groups by HPLC. Data are expressed as mean ± SD (*n* = 5). Figure S5. Lung wet/dry (W/D) weight ratio in different groups. Data are expressed as mean ± SD (*n* = 5). Significance was analyzed using one-way ANOVA with Tukey’s post hoc-test for multiple comparisons; and * *p* < 0.05, ** *p* < 0.01 and *** *p* < 0.001, n.s. indicates not significant. Figure S6. (a) Immunofluorescence staining of neutrophils in lung tissues. (b) Cell populations and quantification of bronchoalveolar lavage fluid cells in different groups. Data are expressed as mean ± SD (*n* = 5), Significance was analyzed using one-way ANOVA with Tukey’s post hoc-test for multiple comparisons; *** *p* < 0.001, n.s. indicates not significant. Figure S7. Serum levels of CRP, TNF-α, IL-6, and IL-10 at 12, 24, 48, and 72 h after treatment. Data are expressed as mean ± SD (*n* = 5). Significance was analyzed using one-way ANOVA with Tukey’s post hoc-test for multiple comparisons; and n.s., not significant (*p* > 0.05). Figure S8. Hematological parameters including HGB, RBC, PLT, and WBC counts at 12, 24, 48, and 72 h after treatment Data are expressed as mean ± SD (*n* = 5). Significance was analyzed using one-way ANOVA with Tukey’s post hoc-test for multiple comparisons; and n.s., not significant (*p* > 0.05). Figure S9. Serum liver function markers (ALT, AST, ALP, and TP) at different time points (12, 24, 48, and 72 h). Significance was analyzed using one-way ANOVA with Tukey’s post hoc-test for multiple comparisons; and n.s., not significant (*p* > 0.05). Figure S10. Serum renal function markers (BUN and CREA) at different time points (12, 24, 48, and 72 h). Significance was analyzed using one-way ANOVA with Tukey’s post hoc-test for multiple comparisons; and n.s., not significant (*p* > 0.05).
