# Supplementary material for: Therapeutic Effects of Intratracheally Nebulized Carnosine-Loaded Liposomes on Lipopolysaccharide-Induced Acute Lung Injury
Source: Pharmaceutics. 2026 Aug 29;18(9):1090. doi: 10.3390/pharmaceutics18091090 (PMC13610148; doi:10.3390/pharmaceutics18091090)
Supplement: Supplementary file 1 [file pharmaceutics-18-01090-s001.zip › pharmaceutics-4421377-supplementary.pdf]

pharmaceutics

MDPI

Supplementary Materials: Therapeutic Effects of Intratracheally Nebulized Carnosine-Loaded Liposomes on Lipopolysaccharide-Induced Acute Lung Injury

Chao Fang, Lixin Xie, Daihan Xie, Jingting Yin, Shiji Zhang, Yiling Gan, Xiaodong Hou, Fanlei Kong, Yu Huo and Xiuli Liu

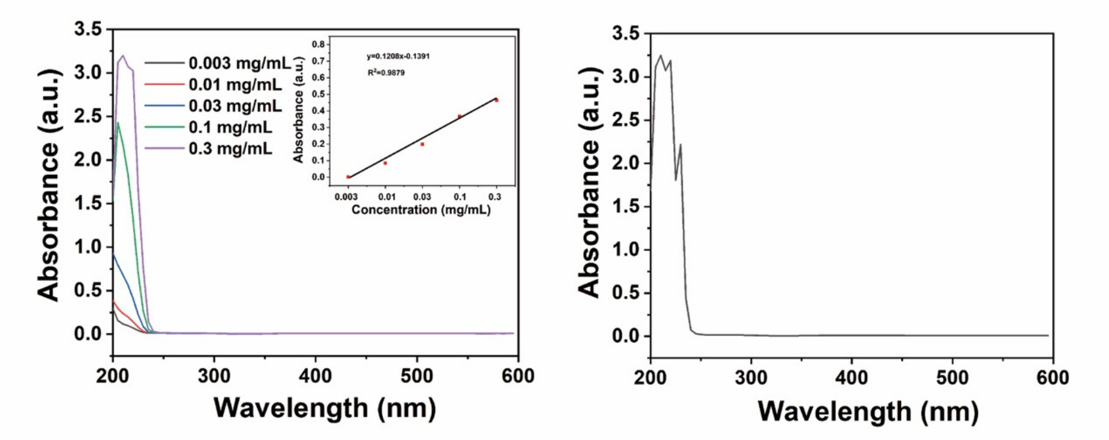

Figure S1. UV-Vis absorption spectra of carnosine at various concentrations (0.003, 0.01, 0.03, 0.1, and 0.3 mg/mL) measured over a wavelength range of 200 – 600 nm. (a) The left panel and its inset show the absorption profiles and the corresponding calibration curve illustrating the linear relationship between the absorbance and carnosine concentration. (b) The right panel displays a representative UV-Vis spectrum of carnosine, highlighting the characteristic absorption peak at approximately 210 nm.

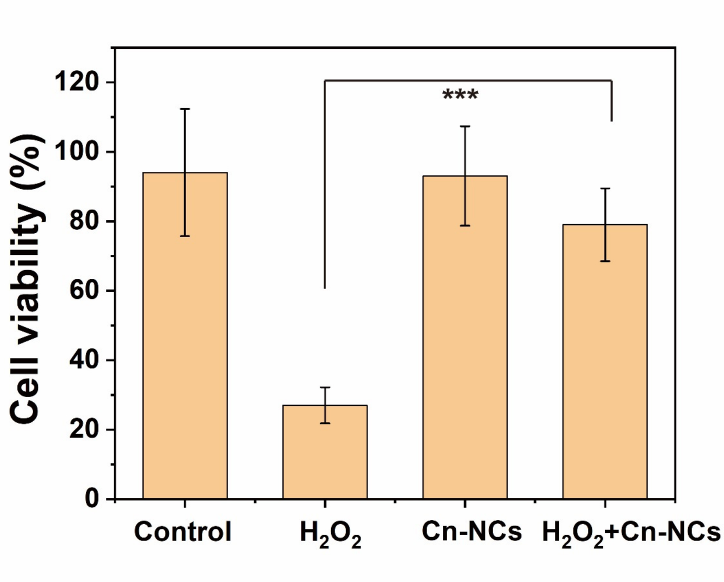

Figure S2. Viability of MLE-12 cells in different groups. Data are expressed as mean ± SD (n = 3). Significance was analyzed using one-way ANOVA with Tukey's post hoc-test for multiple comparisons; and *** $p < 0.001$.

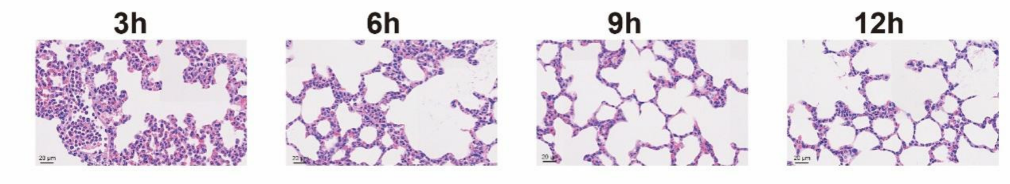

Figure S3. Time-course H&E staining of lung tissues in the Cn-Ls-treated group at 3, 6, 9, and 12 h post-administration (scale bar: 20 μm).

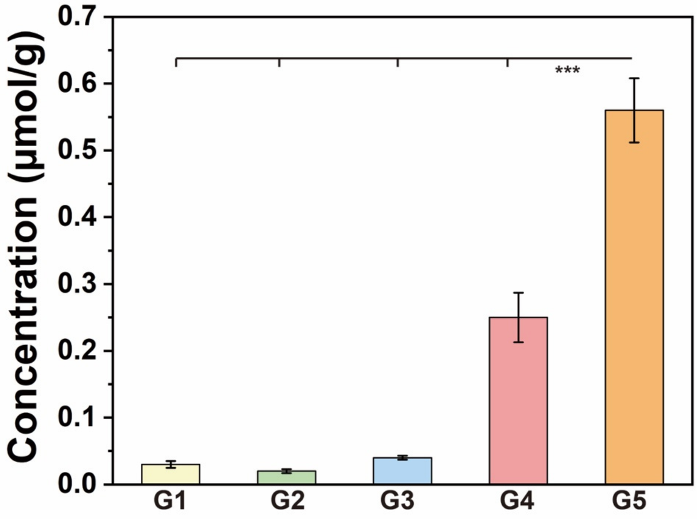

Figure S4. Carnosine levels in lung tissues of different groups by HPLC. Data are expressed as mean ± SD ($n = 5$).

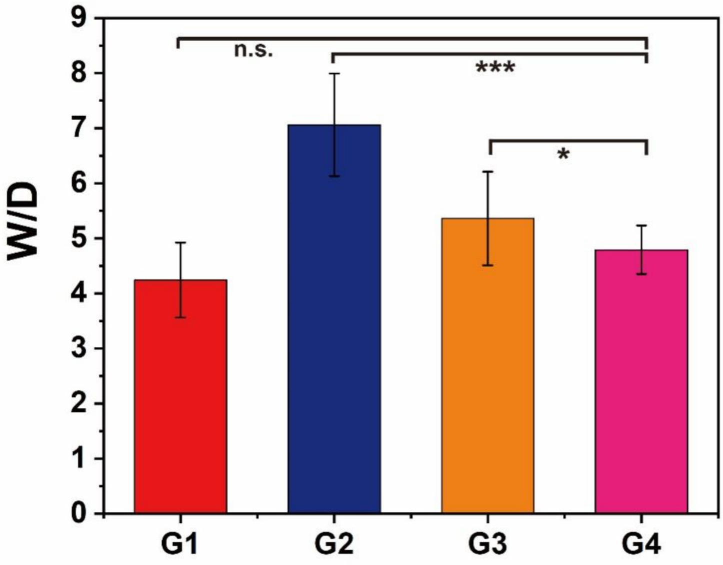

Figure S5. Lung wet/dry (W/D) weight ratio in different groups. Data are expressed as mean ± SD ($n = 5$). Significance was analyzed using one-way ANOVA with Tukey's post hoc-test for multiple comparisons; and * $p < 0.05$, ** $p < 0.01$ and *** $p < 0.001$.

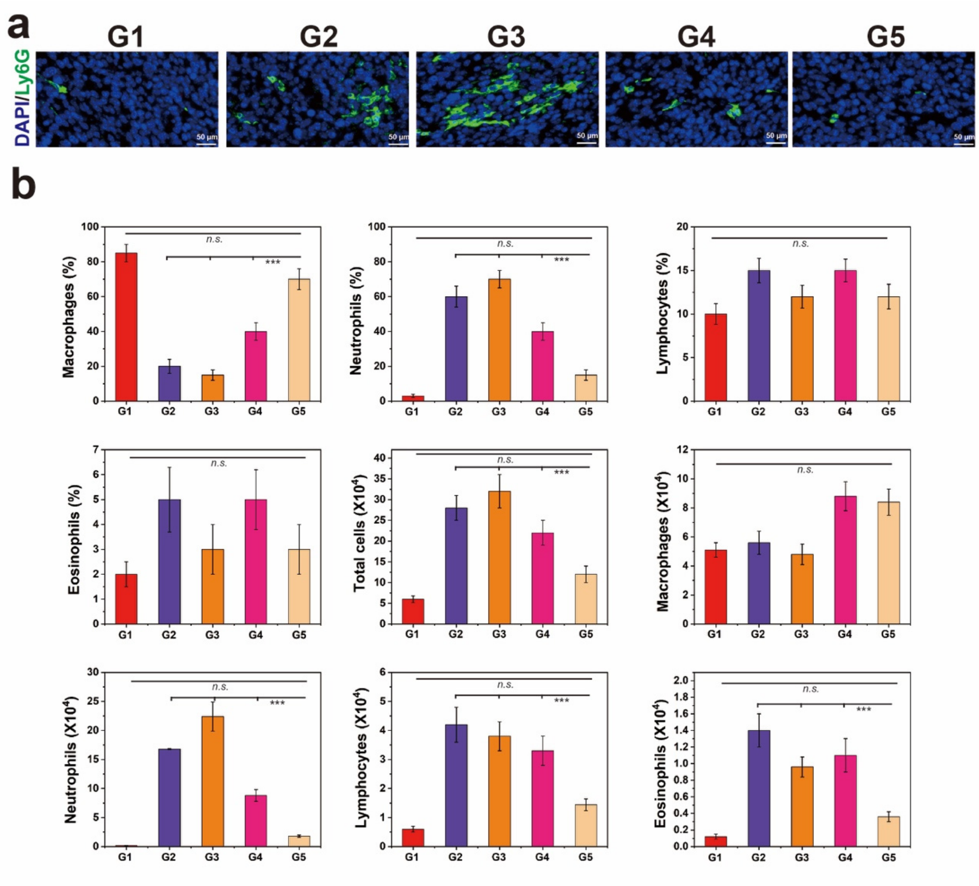

Figure S6. (a) Immunofluorescence staining of neutrophils in lung tissues. (b) Cell populations and quantification of bronchoalveolar lavage fluid cells in different groups. Data are expressed as mean ± SD ($n = 5$), Significance was analyzed using one-way ANOVA with Tukey's post hoc-test for multiple comparisons; and * $p < 0.05$, ** $p < 0.01$ and *** $p < 0.001$.

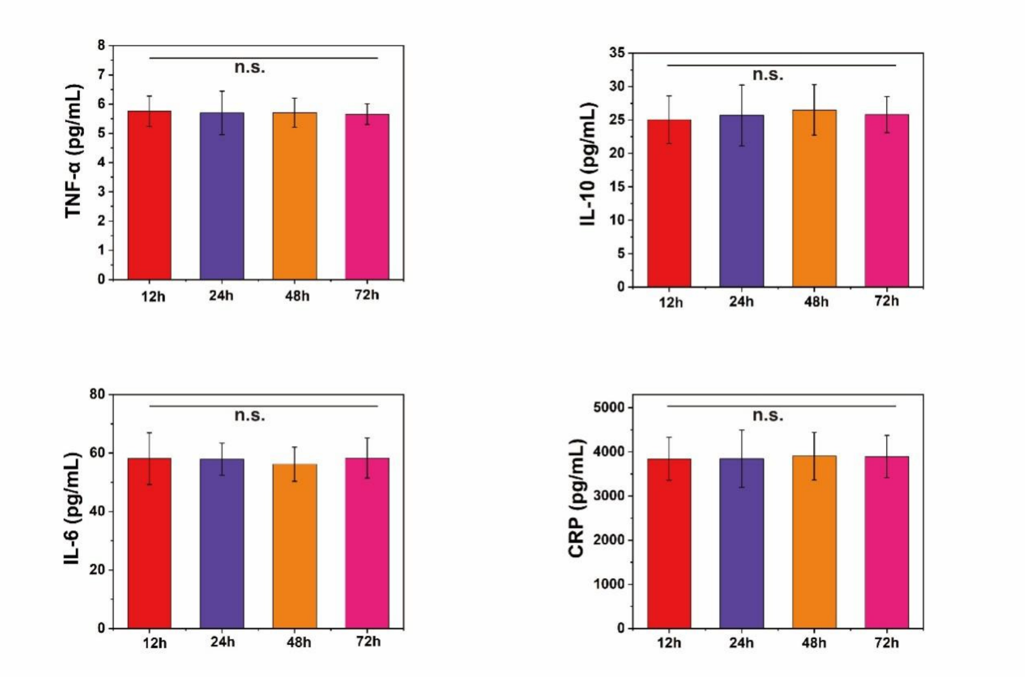

Figure S7. Serum levels of CRP, TNF-α, IL-6, and IL-10 at 12, 24, 48, and 72 h after treatment. Data are expressed as mean ± SD ($n = 5$). Significance was analyzed using one-way ANOVA with Tukey' s post hoc-test for multiple comparisons; and *n.s.*, not significant ($p > 0.05$).

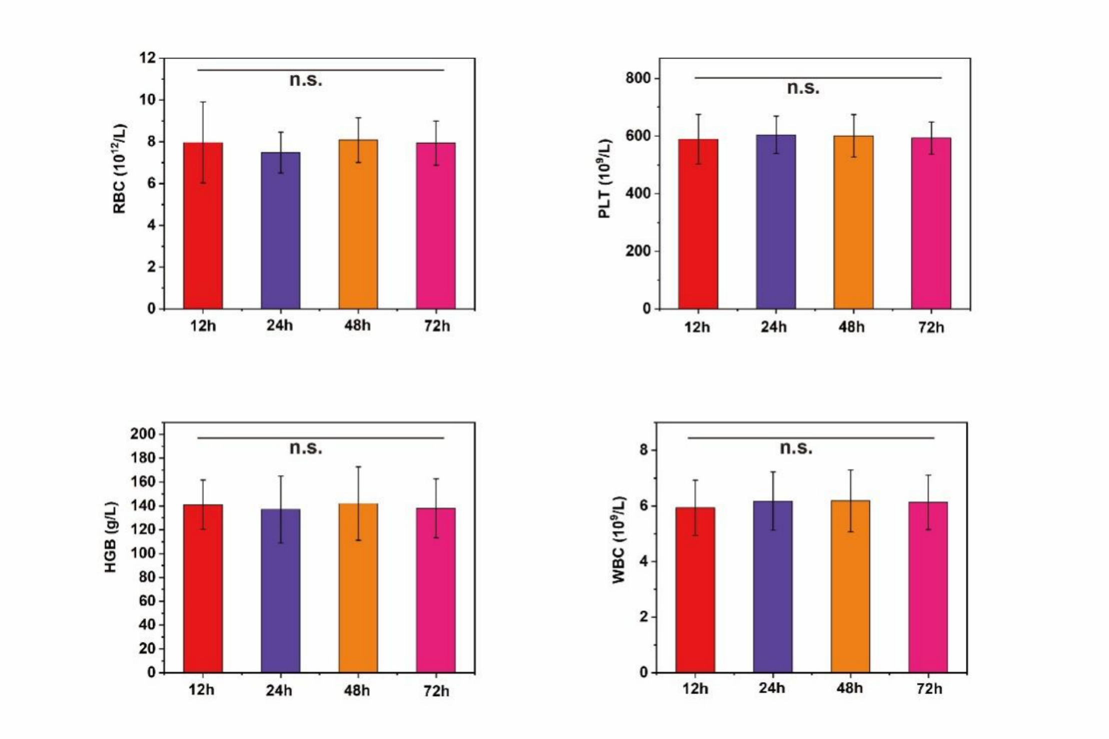

Figure S8. Hematological parameters including HGB, RBC, PLT, and WBC counts at 12, 24, 48, and 72 h after treatment Data are expressed as mean ± SD ($n = 5$). Significance was analyzed using one-way ANOVA with Tukey's post hoc-test for multiple comparisons; and *n.s.*, not significant ($p > 0.05$).

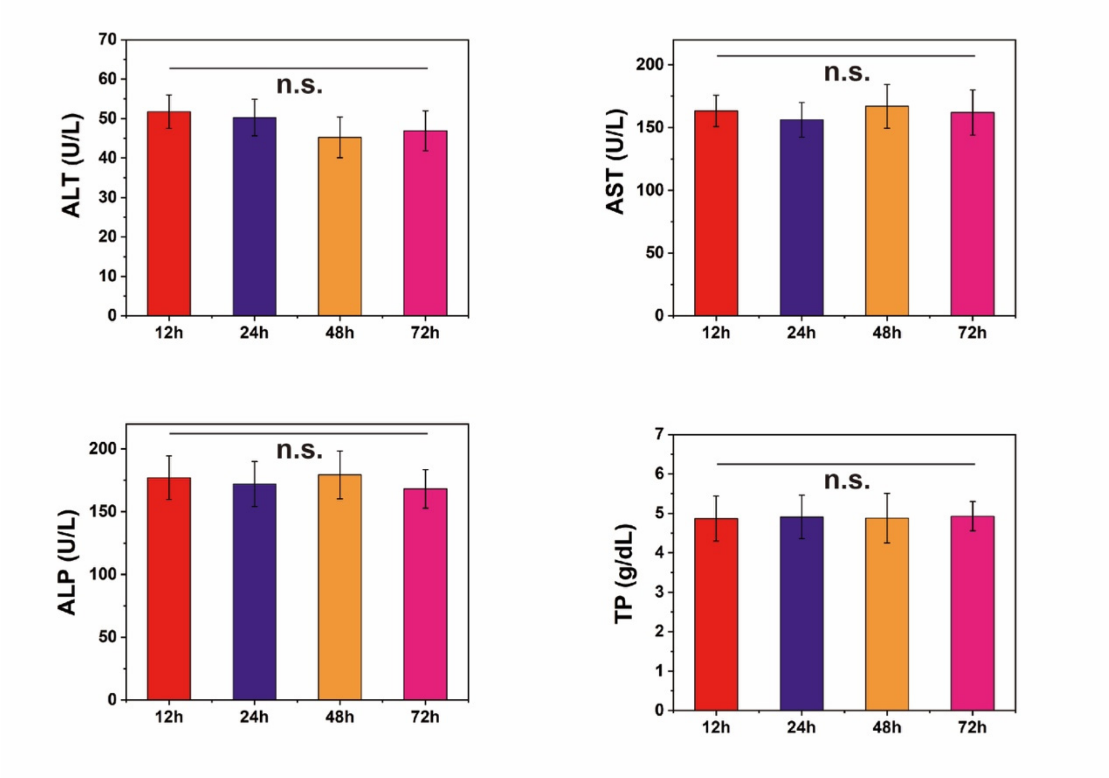

Figure S9. Serum liver function markers (ALT, AST, ALP, and TP) at different time points (12, 24, 48, and 72 h). Significance was analyzed using one-way ANOVA with Tukey's post hoc-test for multiple comparisons; and n.s., not significant ($p > 0.05$).

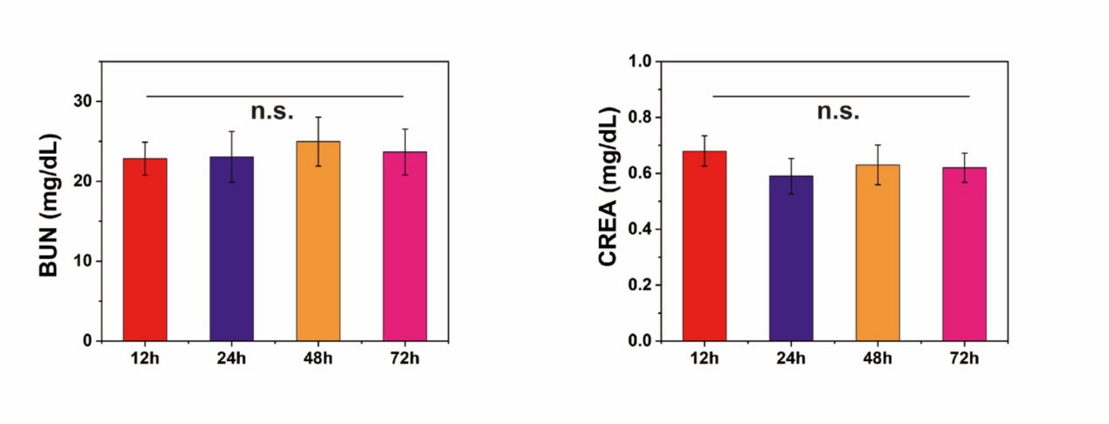

Figure S10. Serum renal function markers (BUN and CREA) at different time points (12, 24, 48, and 72 h). Significance was analyzed using one-way ANOVA with Tukey's post hoc-test for multiple comparisons; and *n.s.*, not significant ($p > 0.05$).
